# Comparative evaluation of crushing methods on the deagglomeration and enhanced properties of reclaimed asphalt pavement

**DOI:** 10.1371/journal.pone.0358448

**Published:** 2026-10-26

**Authors:** Jun Yang, Zhengmao Wei, Bo Li, Tao Chen, Xu Lu, Chengxia Li, Peilin Liu

**Affiliations:** 1 Ningxia Transport Science Research Institute Co., Ltd., Yanchuan, China; 2 Gansu Yuanda Road Industry Group Co., Ltd., Lanzhou, China; 3 Gansu Industry Technology Center of Transportation Construction Materials Research and Application, Lanzhou Jiaotong University, Lanzhou, China; 4 The Highway Development Center of Gansu Province, Lanzhou, China; 5 County and Township Highway Maintenance Station of Jingyuan County, Jingyuan, China; Graphic Era Deemed to be University, INDIA

## Abstract

Crushing treatment is essential for improving reclaimed asphalt pavement (RAP) quality, but how different crushing mechanisms affect aggregate-scale properties remains poorly quantified. Four crushing methods, namely small mesh hammer crushing, hammer crushing, jaw crushing, and double roller crushing, were compared in terms of deagglomeration efficiency and aggregate property enhancement. The residual mass ratio and physical and mechanical properties, including water absorption, crushing value, abrasion value, and needle and flake particle content, were evaluated. The surface micromorphology and roughness of RAP aggregates were examined using scanning electron microscopy (SEM) and 3D laser scanning. Small mesh hammer crushing achieved the highest deagglomeration efficiency, with residual mass ratios of 77.8%–96.6%. Hammer crushing reduced crushing and abrasion values by 6.14% and 23.87%, respectively. Water absorption increased by up to 32.86% in coarse fractions but remained within technical limits. Regression analysis confirmed strong correlations (R² > 0.7) between the residual mass ratio and aggregate properties. These findings provide a mechanistic basis for selecting crushing methods to optimize RAP quality for highway base applications.

## 1. Introduction

As the mileage of roads requiring maintenance increases, the amount of stockpiled waste asphalt milling material also increases rapidly. The disposal of these milling materials consumes substantial land resources and places considerable pressure on the environment. Asphalt recycling technology, based on the concept of a circular economy, facilitates the effective reuse of RAP while substantially reducing road construction costs and the consumption of non-renewable resources. This aligns with the broader global research trend aimed at utilizing recycled waste materials to develop more environmentally friendly and sustainable low-carbon road infrastructure, as extensively reviewed in recent literature [1]. The pursuit of such recycling is driven not only by economic incentives but also by significant environmental benefits across the entire life cycle of pavement materials, as highlighted in recent systematic assessments of its benefits [2]. Recently, Samadi et al. [3] utilized drop-weight impact tests and microstructural analysis to evaluate sustainable concrete composites, verifying that enhancing the internal structural integrity is critical for improving energy absorption and impact resistance. Similarly, Murali et al. [4] provided a comprehensive review of the sustainable transformation of waste materials, concluding that optimizing microstructural characteristics and pore refinement is imperative for ensuring the mechanical strength and durability of recycled infrastructure. Research findings indicate that variability in RAP gradation, deterioration in the mechanical properties and pavement performance of recycled mixtures caused by agglomeration, and RAP contents typically below 30% remain major concerns [5–7].

To improve RAP consistency and its performance in recycled mixtures, researchers have investigated various RAP recycling and processing methods. Sasidharan et al. [8] conducted a characterization study of RAP materials for use in hot-mix asphalt, highlighting methods to assess their variability and suitability. Ding et al. [9] investigated the effects of coarse aggregate morphology parameters on the workability and flowability of asphalt mixtures through a detailed parametric analysis. In a study on the rejuvenation of aged asphalt, Tao et al. [10] evaluated the effectiveness of a surfactant-foaming warm recycling technology. Yao et al. [11] undertook a comprehensive evaluation of the RAP chunks produced during cold milling operations, analyzing their characteristics and the mechanisms behind their formation. Although identifying key sieve sizes, adjusting the gradation design, changing the milling speed, and stockpiling RAP in stages can reduce RAP variability, the methods above proved unsuccessful in effectively stripping the aged asphalt from the surface of the RAP material and separating the aggregate from the asphalt mortar. The use of mechanical equipment for refining RAP material offers several advantages, including cost-effectiveness, high processing efficiency, and a well-established technological foundation. This technological foundation facilitates the decomposition of RAP material agglomerates, thereby enhancing the overall utilization rate. Qiu et al. [12] proposed a refined RAP separation process based on mechanical crushing, and the coarse aggregates treated using this process met the requirements for virgin aggregates. Zaumanis et al. [13] conducted experimental milling investigations and examined the effects of mechanical compression on the interfacial bonding characteristics and bitumen content of RAP. The feasibility of the milling method for separating asphalt binder from aggregate was verified. Li et al. [14] utilized centrifugal crushing equipment and multistage synergistic screening technology to separate RAP materials. The findings demonstrated that the crushing and screening process effectively mitigated the agglomeration of RAP materials, thereby enhancing the stability of the gradation. It is particularly noteworthy that the agglomeration degree of RAP coarse aggregates with particle sizes of 5–10 mm and 10–15 mm was reduced to less than 21.6%. Ai et al. [15] quantitatively analyzed the variability and performance changes of RAP materials during the rotational decomposition process. They also evaluated the laboratory performance of recycled asphalt mixtures with different RAP dosages. The findings demonstrated that the Marshall stability of recycled asphalt mixtures with a RAP material content exceeding 40% following the rotational decomposition process still complied with the specified requirements. Xu et al. [16] developed a flexible-rigid coupled RAP separation technique and compared its effects with those of the conventional plant separation method on the properties of RAP materials and their blends. The homogeneity of the RAP material distribution in the mix was analyzed by the researchers using scanning electron microscopy and CT scanning techniques. The findings indicated that the laboratory separation technique significantly reduced the agglomeration of RAP materials with particle sizes of 10–20 mm. Consequently, this enhanced the homogeneity of the recycled mixtures. However, this method also resulted in a decrease in the dynamic stability of the mixtures. Current research mainly focuses on the effects of pretreatment on the refined separation of RAP and the performance of recycled mixtures. Comparative analyses of the effects of different crushing methods on the physical and mechanical properties and micromorphology of RAP aggregates remain limited, and the relationships among aggregate properties, micromorphology, and agglomeration degree are still unclear.

Although previous studies have shown that mechanical crushing generally improves RAP properties [17–19], current understanding lacks a systematic quantification of how specific crushing mechanisms—namely impact, compression, shear, and attrition—differentially alter the micro-interface of the aggregate-mastic system. To address this gap and the limitations of previous single-method studies, this research implements a comparative investigation utilizing small mesh hammer for impact and size-exclusion, standard hammer for impact, jaw for compression, and double roller for combined compression and shear crushing. Moving beyond simple performance verification, this study explicitly contributes the following insights: first, differentiating the fundamental deagglomeration mechanisms by contrasting impact-induced fracture against compression-induced failure, evaluating their efficiency through the Residual Mass Ratio index; second, utilizing 3D laser scanning and SEM to establish the missing micro-macro linkage between microscopic roughness evolution and macroscopic physical properties; and third, establishing a quantitative regression model to analytically confirm the correlation, with R^2^ > 0.7, between deagglomeration degree and mechanical indicators. This framework quantifies the engineering trade-off between binder removal and aggregate integrity, providing a theoretical basis for optimizing industrial RAP processing. The flowchart of the experimental design is shown in Fig 1.

**Fig 1:**
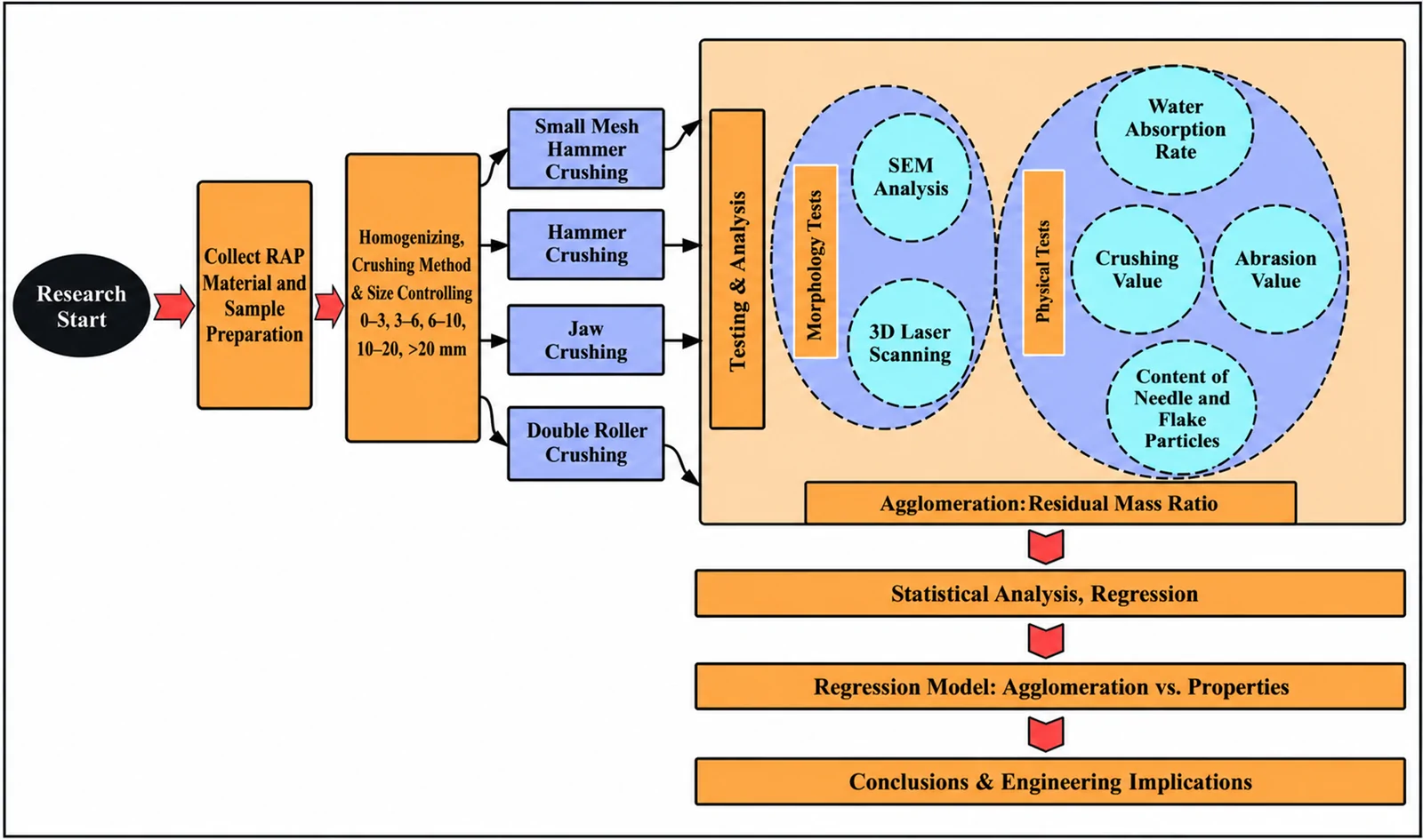
The flowchart of the experimental design.

## 2. Materials

The RAP material used in the test was sourced from the Jingtai to Shuiquan section of the G247 line in Gansu Province, while the natural aggregate selected was limestone. To ensure the representativeness of the samples and minimize variability within the stockpile, a bulk batch of RAP was initially collected and subsequently homogenized using the standard quartering method before testing. The initial asphalt content of this homogenized RAP was determined to be 6.4% utilizing the centrifugal separation method. To evaluate the aging characteristics of the binder, the recovered asphalt was tested in accordance with standard procedures. The results showed a penetration of 24 (0.1 mm at 25°C) and a softening point of 65°C, indicating that the binder had undergone significant oxidative aging and exhibited high stiffness. The physical and mechanical properties of the aggregate were tested in accordance with the prescribed test methods as detailed in the specification. Specifically, the asphalt extraction tests were conducted using an automatic centrifuge extractor model 75-PV50A25 manufactured by Controls in Italy to ensure consistent binder removal. The results of these tests are presented in Tables 1–3.

**Table 1:** RAP material and natural aggregate mud content.

Mud Content	Particle Size				Technical Requirement	Test Method
Particle size range (mm)	5 ~ 10	10 ~ 15	15 ~ 20	20 ~ 25	≤1.2	T 0310
RAP (%)	0.96	0.70	0.73	0.75		
Limestone (%)	0.39	0.14	0.24	0.33		

**Table 2:** Needle-flake particle content of RAP material and natural aggregate.

Needle-flake particle content	Particle Size				Technical Requirement	Test Method
Particle size range (mm)	5 ~ 10	10 ~ 15	15 ~ 20	20 ~ 25	≤18	T 0312
RAP (%)	2.89	4.15	7.73	9.28		
Limestone (%)	4.97	5.83	11.06	11.49		

**Table 3:** RAP material and natural aggregate crushing value.

Crushing Value	Test number			Average Value (%)	Technical Requirement	Test Method
	1	2	3			
RAP (%)	21.2	19.2	20.8	20.5	≤22	T 0316
Limestone (%)	15.9	16.1	18.4	17.5		

## 3. Methodology

### 3.1 RAP material crushing and extraction test

To reduce the agglomeration of the raw RAP material, four methods were used: small mesh hammer crushing, hammer crushing, jaw crushing, and double roller crushing.

The crushing experiments utilized three distinct laboratory-scale devices to simulate different mechanical actions. Hammer crushing was performed using a device manufactured by Henan Longyuan Machinery Co., Ltd. in Zhumadian, China. This crusher operated at a high rotor speed of 1300 rpm. Two specific configurations were employed: the standard setup used a 40 mm aperture screen and a processing time of approximately 30 s, whereas the small mesh setup used a 25 mm aperture screen and a processing time of approximately 50 s to intensify the shearing and grinding effects. Jaw crushing was conducted using equipment developed by Hebei Fengyuan Experimental Instrument Manufacturing Co., Ltd. in Hebei, China, with an eccentric shaft speed of 280 rpm. The discharge opening was standardized at a closed side setting of 25 mm. This specific setting was selected to ensure that the target maximum particle size was consistent with the aperture of the small mesh hammer configuration, thereby facilitating a comparable evaluation of the crushing mechanisms. Finally, double roller crushing was performed using a machine produced by Shandong Huaji Heavy Industry Co., Ltd. in Weifang, China. This device utilized counter-rotating rolls with a gap setting of 10 mm and a rotational speed of 153 rpm, with a crushing duration maintained at 60 seconds. These specific parameters were selected to represent distinct dominant crushing mechanisms, contrasting the extrusion force of jaw and roller crushers against the impact and shear forces of the hammer crusher.

The operational parameters for the four crushing methods were selected based on standard laboratory equipment specifications to simulate typical mechanical actions. Specifically, the rotor speed for the hammer crushers was maintained at 1300 rpm to provide sufficient impact energy. The gap setting for the double roller crusher was fixed at 10 mm, and crushing durations ranging from 30 to 60 seconds were established. However, it is essential to acknowledge that these laboratory-scale devices cannot fully replicate the continuous stress states, high-throughput dynamics, and complex particle-particle interactions encountered in industrial-scale RAP processing plants. While the fundamental fracture mechanics, including impact, compression, and shear, are preserved at the laboratory scale, the discrete batch processing differs from the continuous particle-bed interactions of industrial operations. Therefore, these operational parameters balance deagglomeration efficiency with aggregate preservation under controlled laboratory conditions.

In this study, the pretreated RAP was divided into five particle size fractions: 0–3 mm, 3–5 mm, 5–10 mm, 10–20 mm, and 20–25 mm. To accurately determine the residual mass ratio, the aged asphalt binder was first quantitatively extracted from RAP materials of different particle sizes following the JTG E20-2011 T0722 standard, using trichloroethylene as the solvent. This ensured complete binder separation. Then, the residual mass on each corresponding sieve was measured in three replicates, both before and after extraction. To quantitatively evaluate the effect of crushing on the agglomeration degree of RAP materials of varying particle sizes, the residual mass ratio was introduced as an evaluation index. Residual mass ratio refers to the proportion of the sieve mass of the aggregates remaining in the sieve after extraction of RAP material to the sieve mass of the aggregates extracted before extraction, expressed by P. The larger P is, the lower the degree of agglomeration of the RAP material, as shown in equation (1):


$$\begin{array}{c} P=\frac{r_{\text{1}}}{r_{\text{2}}}\times 100 \end{array}$$ (1)

Where: P is the residual mass ratio of RAP material in the grade, %. *r*_1_ is the mass of aggregate remaining on the sieve of the grain size after extraction, g. $r_{\text{2}}$ is the mass of RAP material on the sieve of the grain size prior to extraction, g.

Justification of the Evaluation Index: Physically, the residual mass ratio P serves as a quantitative proxy for the deagglomeration efficiency, reflecting the proportion of RAP structures that remain stable as individual aggregates rather than disintegrating into finer fractions upon binder removal. A higher P value indicates that the crushing process has effectively stripped the binding mastic, leaving behind structural aggregates comparable to the extracted gradation. However, it must be acknowledged that this index has limitations: as a mass-based geometric parameter, it assumes relative homogeneity in aggregate density and does not fully decouple the influence of the aged binder’s rheological stiffness from the mechanical interlocking of the agglomerates. Nevertheless, compared to established volumetric methods such as X-ray CT, which are limited by sample size, this index provides a scalable industrial validation, corroborated by its strong correlation (R^2^ > 0.7) with mechanical performance indicators in subsequent analyses.

### 3.2 Physical and mechanical properties of RAP material

#### 3.2.1 Water absorption rate.

The water absorption of RAP and natural aggregate before and after crushing was determined in accordance with the ‘Aggregate Testing Procedure for Highway Engineering’ (JTG 3432−2024) and calculated using equation (2).


$$\begin{array}{c} \omega_{x}=\frac{m_{f}-m_{a}}{m_{a}}\times 100 \end{array}$$ (2)

Where: $\omega_{x}$ is the water absorption rate of the aggregate, %. $m_{f}$ is the surface dry mass of the aggregate, g. $m_{a}$ is the dry mass of the aggregate, g.

#### 3.2.2 Crushing value.

The crushing values of RAP material and natural aggregate before and after crushing were determined in accordance with the ‘Aggregate Testing Procedure for Highway Engineering’ (JTG 3432−2024) and calculated using equation (3).


$$\begin{array}{c} \text{ACV}=\frac{m_{\text{2}}}{m_{\text{1}}+m_{\text{2}}}\times 100 \end{array}$$ (3)

Where: ACV is the aggregate crushing value, %. $m_{\text{2}}$ is the mass under the 2.36 mm sieve, g. $m_{\text{1}}$ is the mass over the 2.36 mm sieve, g.

#### 3.2.3 Abrasion value.

The abrasion value of RAP material and natural aggregate before and after crushing was determined according to the ‘Aggregate Testing Procedure for Highway Engineering’ (JTG 3432−2024), and the abrasion value was calculated according to equation (4).


$$\begin{array}{c} \text{LA=}\frac{m_{\text{1}}-m_{\text{2}}}{m_{\text{1}}}\times 100 \end{array}$$ (4)

Where: LA is the aggregate abrasion value, %. $m_{\text{1}}$ is the mass of the sample before the test, g. $m_{\text{2}}$ is the mass retained on the 1.7 mm sieve, g.

#### 3.2.4 Content of needle and flake particles.

The needle and flake particle content of RAP and natural aggregate before and after crushing was determined in accordance with the ‘Aggregate Testing Procedure for Highway Engineering’ (JTG 3432−2024) and the needle and flake particle content was calculated according to formula (5).


$$\begin{array}{c} Q_{e\&f}=\frac{m_{\text{1}}}{m_{\text{1}}+m_{\text{2}}}\times 100 \end{array}$$ (5)

Where: $Q_{e\&f}$ is the content of aggregate needles and flakes, %. $m_{\text{1}}$ is the mass of needles and flakes, g; $m_{\text{2}}$ is the mass of non-needles and flakes, g.

### 3.3 Microstructure testing of RAP materials

#### 3.3.1 SEM-based micro-morphological testing.

In this paper, the microstructure of natural aggregates and RAP materials was observed using SEM, and the effect of crushing on the micro-morphology of RAP materials was analyzed. Five specimens from each of the limestone, RAP after hammer crushing, and uncrushed RAP groups were selected, each with a sample diameter of approximately 35 mm. The samples were then subjected to a sequential cleaning and drying process to ensure the structural integrity of the aggregate surface. Following this, the samples were affixed to a metal sample stage using conductive adhesive to enhance electrical contact. In order to enhance the electrical conductivity of the samples, Au thin films were deposited on the sample surface using an ion sputter coater. A high-energy electron beam was generated using an electron gun, which was utilized to scan the beam through a grating. This process was repeated line by line, thereby scanning the surface of the sample. The electron beam interacted with the sample atoms, thereby exciting signals such as secondary electrons and backscattered electrons. The detection system acquired and synchronized these signals in real time and converted them into grayscale images based on their intensity differences, thereby generating a microscopic image of the sample. It must be recognized that RAP materials possess inherent spatial heterogeneity. With only five representative particles analyzed per group, namely limestone, RAP after hammer crushing, and uncrushed RAP, these SEM observations provide qualitative visual evidence of surface micro-morphology rather than a statistically representative characterization of the entire material population. The SEM findings were therefore used to corroborate the physical mechanisms inferred from the macroscopic bulk property tests, and should not be interpreted as standalone statistical evidence.

#### 3.3.2 Roughness testing based on 3D laser plane scanner.

In this paper, crushed RAP material, raw RAP material, and limestone aggregate were classified into four grain size intervals ranging from 5 to 25 mm. Five parallel tests were conducted for each grain size to ensure statistical reliability. The surface texture data were acquired using a high precision 3D laser scanning system supplied by Xi’an Zhongzhi Jianwu IoT Technology Co., Ltd., and equipped with a MICRO-EPSILON sensor. This device operated with a vertical measurement precision of 0.01 mm to ensure accurate capture of topographic features. Regarding the data processing, this study did not employ specific digital filtering algorithms to mathematically separate bitumen residue from the natural aggregate texture. Instead, the analysis relied on the physical mechanism by which the asphalt mastic film typically fills the surface voids of the aggregate and reduces its textural depth. Consequently, the arithmetic mean deviation of the surface profile (R_a_) was adopted as the quantitative roughness metric. To account for the inherent heterogeneity of the material, the reported roughness values represented the average of five independent parallel measurements, ensuring that the measurements captured the representative surface characteristics of the processed RAP batch. The complete scanning test and data processing workflow is illustrated in Fig 2.

**Fig 2:**
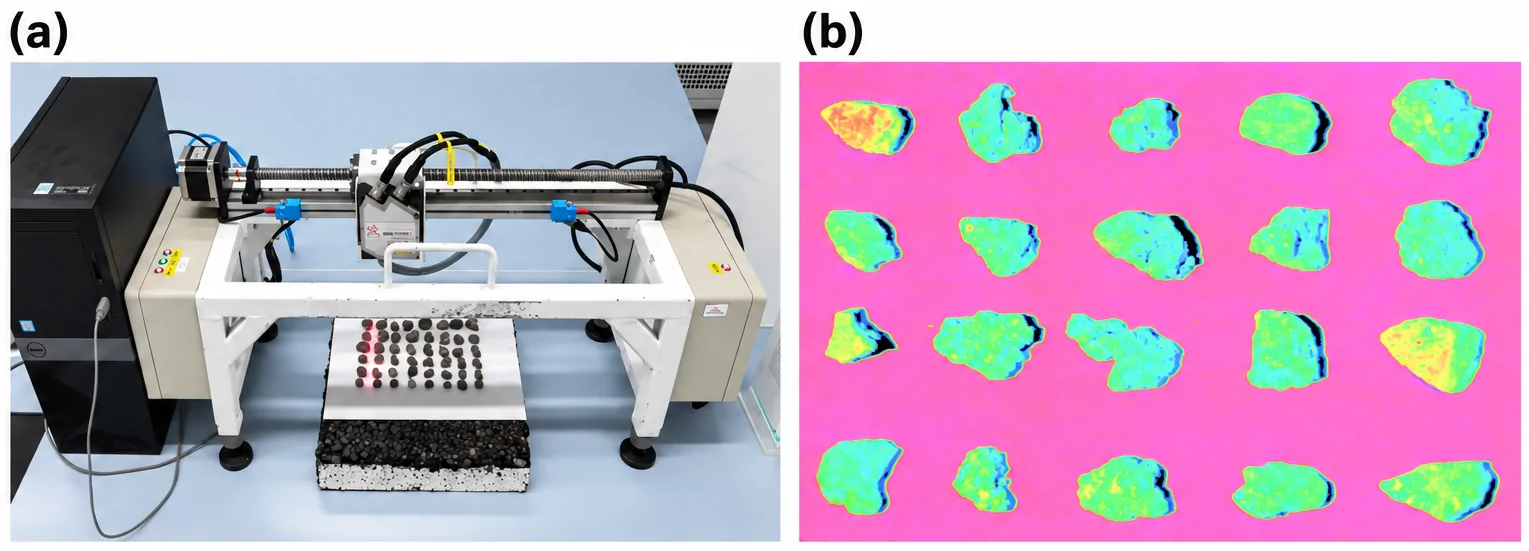
Roughness test. (a) Scanning process. (b) Point cloud processing.

### 3.4 Statistical analysis

To ensure the statistical reliability of the experimental data, the extraction tests and physical property tests (including water absorption, crushing value, abrasion value, and needle and flake particle content) were performed in triplicate (n = 3) for each sample group. The roughness tests utilizing 3D laser scanning were conducted on five replicates (n = 5) for each particle size and crushing method to account for the surface heterogeneity of the RAP materials. In all figures, the error bars represent the standard deviation (SD).

## 4. Results and discussion

### 4.1 Effect of crushing on the degree of agglomeration of RAP material

The residual mass ratio of RAP material with different particle sizes was determined before and after crushing. The test results are shown in Fig 3.

**Fig 3:**
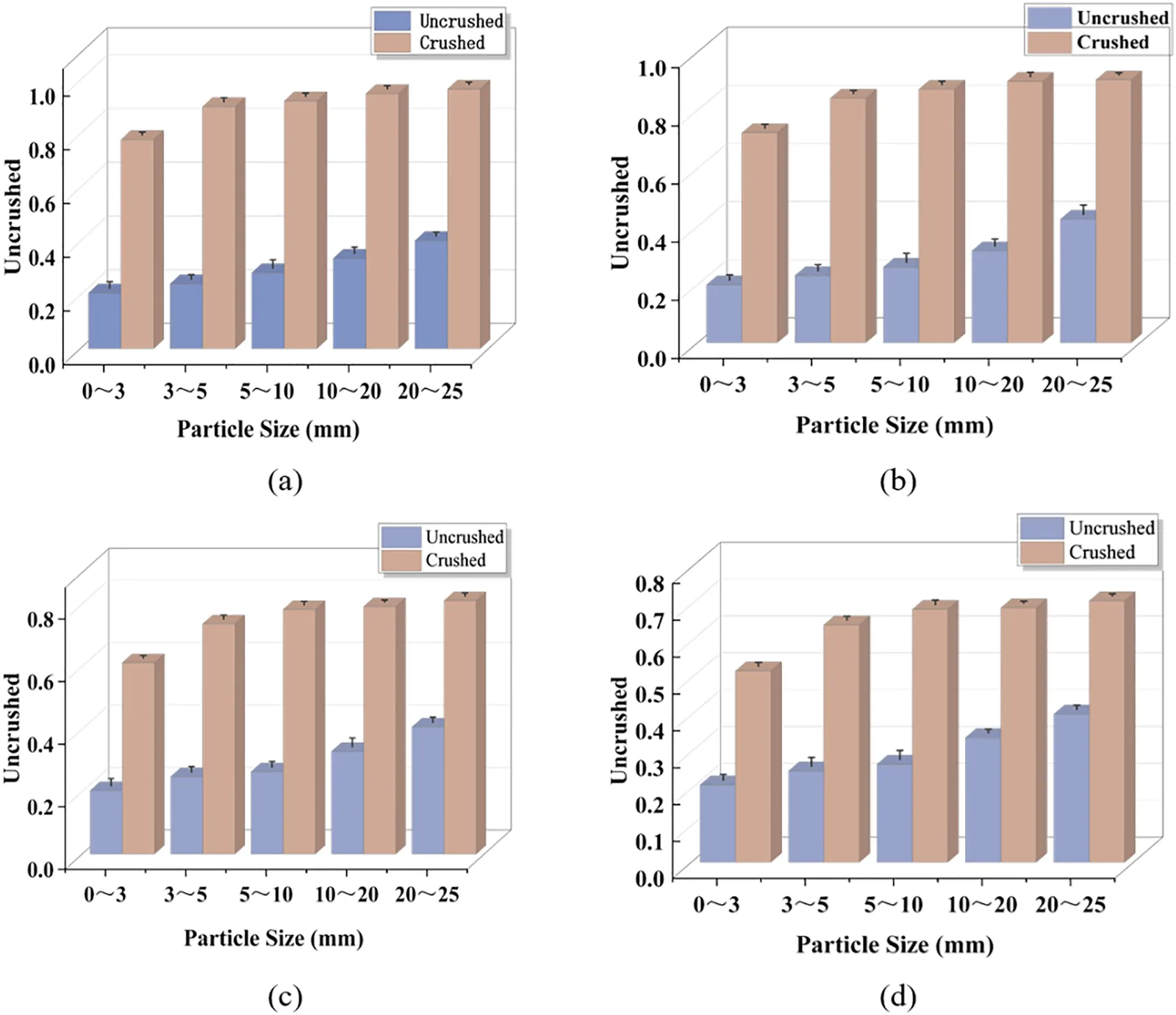
Residual mass ratio of RAP material before and after crushing (a) Small mesh hammer crushing (b) Hammer crushing (c) Jaw crushing (d) Double roller crushing.

The experimental data in Fig 3(a)–3(d) show that all four crushing processes were effective in dissociating RAP agglomerates. The residual mass ratio after treatment increased by more than 75.7% compared to the untreated specimen. In the unfragmented state, the residual mass ratio ranged from 19.9% to 42.5%, and tended to increase with particle size. This indicates that the fine-grained RAP has a stronger tendency to agglomerate. After crushing treatment, the residual mass ratio increased to 56.9%–96.6%, and the crushing effect was positively correlated with the particle size. This phenomenon can be attributed to the fact that small RAP particles can pass directly through the screen openings or gaps in the crushing equipment, resulting in a relatively low degree of agglomerate dissociation. Large RAP particles, on the other hand, can be more adequately crushed by mechanical action, resulting in a significant reduction in agglomerate content.

From Fig 3(a), it can be observed that the range of residual mass ratio of RAP material after hammer crushing with a small mesh was from 77.8% to 96.6%. From Fig 3(b), it can be obtained that the residual mass ratio after hammer crushing ranged from 72.2% to 90.4%. From Fig 3(c), it can be concluded that the range of residual mass ratio after jaw crushing was from 61.2% to 80.0%. From Fig 3(d), it can be concluded that the range of residual mass ratio after double roller crushing was 51.9% to 70.9%. This indicates that small mesh hammer crushing provided the greatest degree of deagglomeration. Without the use of a small screen, the machinery was able to fully decompose the large particle size RAP material agglomerates. However, smaller RAP particles passed directly through the screen without further crushing because of the lack of additional screening and grinding; therefore, the deagglomeration effect was weaker than that of small mesh hammer crushing. Jaw crushing and double roller crushing stripped aged asphalt from the aggregate surface. However, small RAP particles and newly generated fines re-adhered to form agglomerates after compression, resulting in a lower residual mass ratio than that obtained after hammer crushing and small mesh hammer crushing [20].

It is also worth noting that despite the high rotor speed of 1300 rpm employed in hammer crushing, no thermal re-agglomeration of the RAP particles was observed. The particle size distribution data confirm effective deagglomeration rather than clumping. This indicates that the short residence time and the cooling effect of the high-speed airflow effectively prevented the aged binder, which typically possesses a high softening point, from reaching a viscous flow state during processing. Small mesh hammer crushing consistently exhibited the highest mean values with low standard deviations across replicates, verifying its superior stability compared to the higher variability observed in extrusion-based methods.

The observed differences in deagglomeration efficiency can be attributed to the fundamentally different fracture mechanics governing each method. Hammer-based crushing, including both standard and small mesh configurations, relies on high-velocity impact, where kinetic energy transferred to the RAP particles upon collision preferentially propagates cracks along the weakest interfaces—namely, the aged asphalt mastic bonds. This impact-induced fragmentation achieves efficient deagglomeration. In contrast, jaw crushing applies compressive stress, inducing tensile failure only when the internal stress exceeds the aggregate’s tensile strength. Double roller crushing operates through a combination of compression and shear, where the dominant failure mode includes inter-particle attrition. Furthermore, the small mesh configuration introduces a size-exclusion mechanism: particles remaining above the 25 mm mesh are retained and repeatedly impacted, achieving the highest agglomerate dissociation, whereas standard hammer configurations lack this prolonged screening effect.

### 4.2 Effect of crushing on the physical and mechanical properties of RAP materials

#### 4.2.1 Change of water absorption rate.

The test results of the water absorption rate of RAP material with different grain sizes before and after crushing, and the water absorption rate of natural aggregate with different grain sizes are shown in Fig 4(a)–4(d).

**Fig 4:**
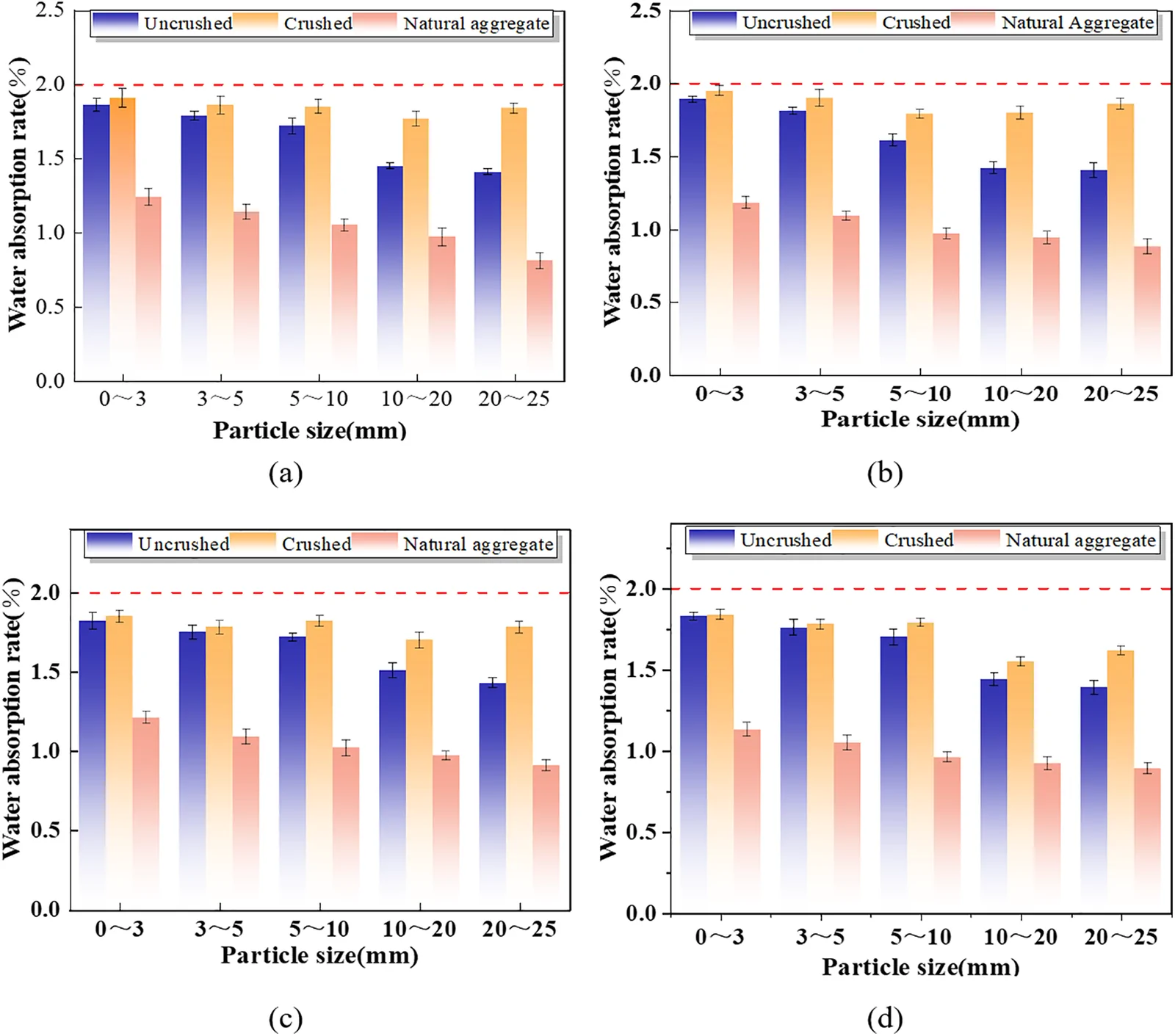
Changing law of water absorption rate of aggregates of various grain sizes after crushing (a) Hammer crushing (b) Small mesh hammer crushing (c) Jaw crushing (d) Double roller crushing.

As shown in Fig 4(a), the water absorption of RAP before and after hammer crushing remained below 2%, and the treated RAP met the applicable technical requirements [21]. The water absorption of RAP before and after crushing was higher than that of natural aggregate, indicating that RAP absorbed more water than natural aggregate. This is because more micro-cracks and pores are created on the surface of the RAP material after processing, resulting in more water entering the RAP material. From Fig 4(a)–4(d), it can be seen that the water absorption ranges after hammer crushing, small mesh hammer crushing, jaw crushing, and double roller crushing were 1.77%–1.91%, 1.79%–1.95%, 1.70%–1.85%, and 1.55%–1.84%, respectively. This indicates that the aggregate has the lowest water absorption after double roller crushing and the highest after small mesh hammer crushing. This is because small mesh hammer crushing removed the most asphalt film compared to other crushing methods [22]. Consequently, the aggregate surface exposes the most microcracks and pores. In contrast, the other three pretreatment methods, particularly double roller crushing, are less effective in stripping the binder, where the retained asphalt film effectively seals the surface pores and inhibits water intrusion.

Comparing Fig 4(a)–4(d), it can be found that the water absorption rate of each grain size of RAP material shows an increasing trend after processing by different crushing methods. This indicates that the refined processing removes the surface asphalt film and increases the aggregate cracks, making it easier for water to enter the pores. The water absorption of each RAP fraction was greater than that of the corresponding natural aggregate. This difference occurred because natural aggregate had a denser surface and fewer pores and microcracks than RAP, making it more difficult for water to penetrate the aggregate. Therefore, natural aggregate had lower water absorption than RAP. As the particle size increased, the water absorption of uncrushed RAP and natural aggregate decreased because, at the same mass, larger particles had a smaller total surface area and a smaller relative contact area with water [23].

The experimental findings presented in Fig 5 demonstrate that the small mesh hammer crushing process has the most substantial influence on enhancing water absorption in RAP materials across all grain sizes. The increases in water absorption for the five particle size fractions were 3.17%, 4.97%, 11.18%, 26.76%, and 32.86%, respectively. This pattern indicates that small mesh hammer crushing is the most effective method of removing asphalt film. In addition, it has been demonstrated that this process can induce micro-cracks and pore structures in the aggregate surface. The findings of the study demonstrate that the water absorption rate of fine aggregate exhibits an increase ranging from 0.55% to 4.97%, while the coarse aggregate demonstrates an increase ranging from 5.29% to 32.8%. These results suggest that the application of fine processing technology leads to a more pronounced enhancement in the water absorption characteristics of coarse aggregate. This phenomenon is attributed to the fact that the crushing process exerts a more significant effect on the dissociation of large-sized RAP agglomerates, while the fine aggregate effectively hinders the penetration of water into the interior of the aggregate due to the retention of a higher content of asphalt film.

**Fig 5:**
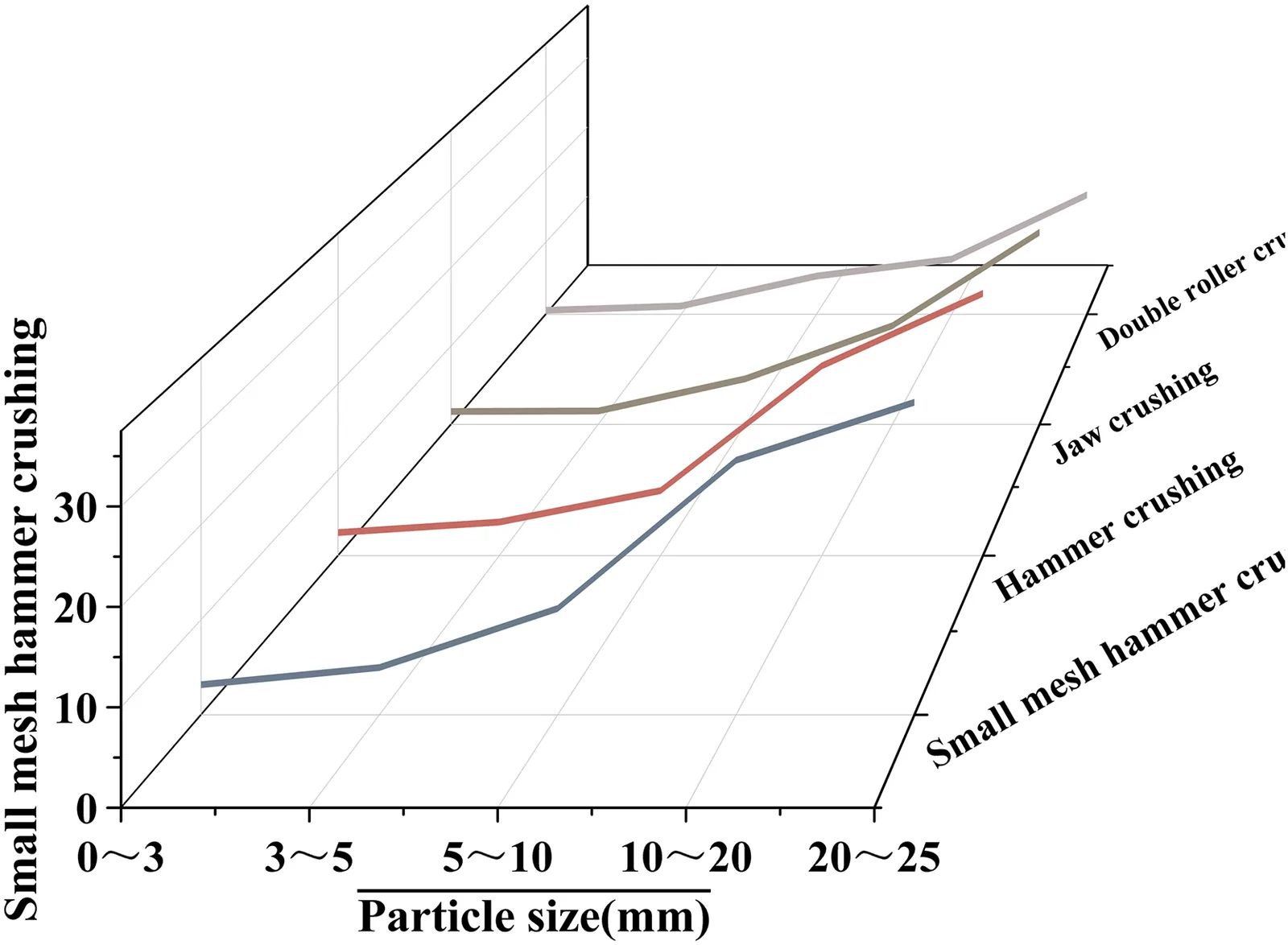
Growth rate of water absorption of RAP materials of various particle sizes after crushing.

The water absorption showed a relative increase of up to 32.86% for coarse aggregates treated by small mesh hammer crushing. This trend indicates both effective asphalt stripping and the formation of micro-cracks. This property has critical implications for practical engineering: higher water absorption implies a higher optimum bitumen content in mix design to compensate for binder absorption. Furthermore, excessive porosity could potentially compromise freeze-thaw durability in cold regions. However, since all measured absolute water absorption values remained below 3%, the processed aggregates satisfied the technical requirements for base layers, effectively balancing deagglomeration benefits with acceptable durability risks.

#### 4.2.2 Change of crushing value.

Fig 6 shows the changes in the crushing value of RAP.As can be seen from Fig 6, the average crushing values of the untreated RAP and natural aggregate were 20.07% and 16.69%, respectively, and both values were below 22%. This shows that the mechanical properties of the untreated RAP material were poorer than those of the natural aggregate, but the crushing value met the technical requirements of base layer construction. Incorporating untreated RAP into the base layer mixture will lead to a reduction in the overall compressive strength of the material. This is because asphalt and fine aggregates adhered to the surfaces of RAP aggregates to form agglomerates. In addition, some aggregates were crushed again during the production of the original asphalt concrete, making the RAP more susceptible to crushing under load. Under external loading, detachment of the asphalt film, decomposition of RAP agglomerates, and fracture of some aggregates caused the crushing value of RAP to be higher than that of natural aggregate. Specifically, the absolute crushing values after treatment were determined to be 18.96% for small mesh hammer crushing, 18.84% for hammer crushing, 19.16% for jaw crushing, and 19.25% for double roller crushing. These values correspond to reductions of 5.52%, 6.14%, 4.52%, and 4.11%, respectively. The crushing values of waste asphalt mixtures after processing by the four crushing methods were still higher than those of natural aggregates. This indicates that the refined processing technology is conducive to improving the load-bearing capacity of RAP materials, and the mechanical properties of recycled asphalt pavement materials are comparable to those of new aggregates after pretreatment. Hammer crushing produced the greatest reduction in crushing value, and double roller crushing was the least effective in improving the mechanical properties of RAP. All four processing methods broke down RAP agglomerates, and the asphalt and fine aggregate were largely removed from the aggregate surface after hammer crushing. Hammer crushing of RAP material using a small mesh produced more cracks in the stone than without using a small mesh, resulting in a lower rate of reduction in crushing value for hammer crushing with a small mesh than for hammer crushing without a small mesh. Jaw crushing and double roller crushing were less effective in reducing the asphalt content of the aggregate and less effective in improving the mechanical properties of the RAP material [24].

**Fig 6:**
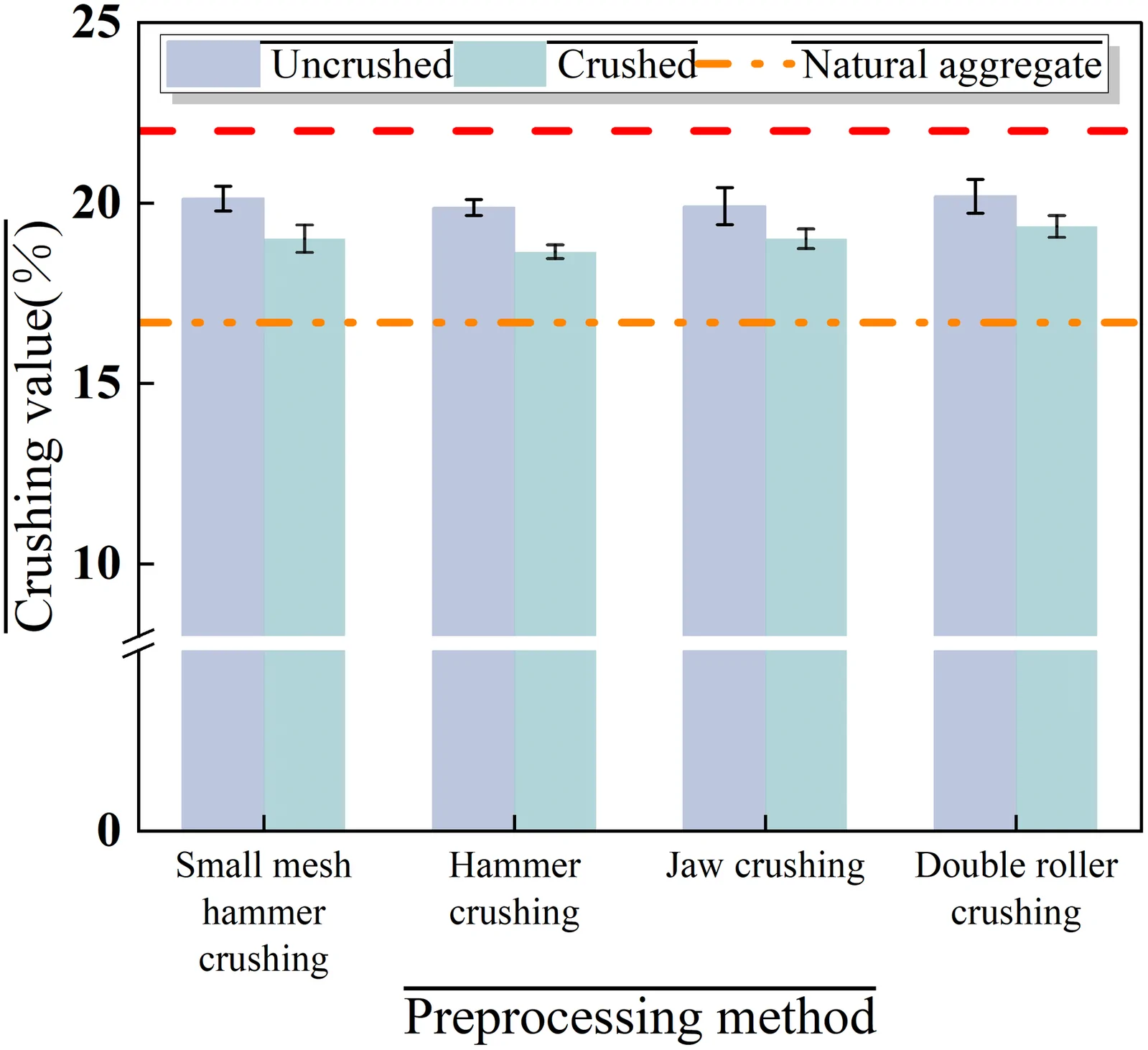
Changing law of crushing value of RAP material after crushing.

#### 4.2.3 Change of abrasion value.

The test results of the change in abrasion value after crushing are shown in Fig 7(a)–7(d).

**Fig 7:**
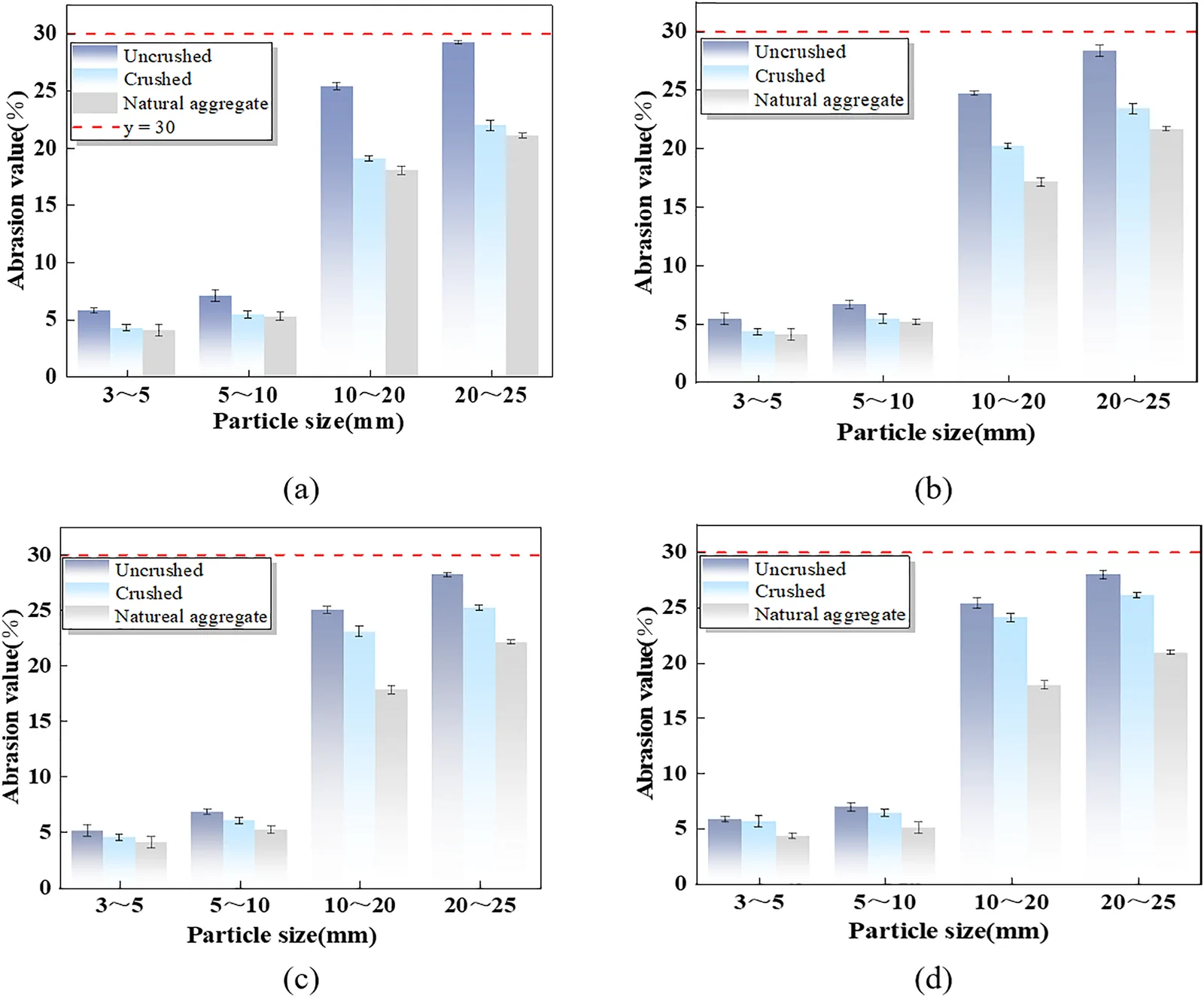
Changing law of abrasion value of aggregates of various grain sizes after crushing (a) Hammer crushing (b) Small mesh hammer crushing (c) Jaw crushing (d) Double roller crushing.

As shown in Fig 7(a), the abrasion values of all RAP particle size fractions before and after hammer crushing were below 30%, and the treated RAP met the technical requirements [25]. The abrasion values of RAP before and after crushing were higher than those of natural aggregate because RAP contained more impurities, such as soil and dust, whereas natural aggregate had a denser and harder surface. Consequently, RAP experienced greater mass loss during the abrasion test. As shown in Fig 7(a)–7(d), the abrasion values after hammer crushing, small mesh hammer crushing, jaw crushing, and double roller crushing ranged from 4.23% to 21.97%, 4.31% to 23.39%, 4.53% to 25.17%, and 5.63% to 26.07%, respectively. RAP had the highest abrasion values after double roller crushing and the lowest after hammer crushing. Hammer crushing opened the greatest number of RAP agglomerates and removed most of the surface dust and clay, whereas the other three methods were less effective in decomposing RAP agglomerates and therefore produced higher abrasion values.

From Fig 7(a)–7(d), it can be seen that the abrasion values of RAP materials of all grain sizes show a decreasing trend after processing by different crushing methods. This indicates that the refined processing removed the asphalt film, fine aggregates, and impurities from the surfaces of coarse aggregates, making the particles harder and denser and improving the abrasion resistance of RAP. The abrasion value of RAP in each particle size fraction was higher than that of natural aggregate because natural aggregate had a denser surface and contained fewer impurities, such as dust, soil, and asphalt. Consequently, the mass loss of natural aggregate during the abrasion test was lower than that of RAP, resulting in a lower abrasion value. As the aggregate particle size increased, the abrasion values of both RAP and natural aggregate increased. This was because, at the same mass, larger particles were subjected to greater impact and friction during the abrasion test, causing the abrasion values of both RAP and natural aggregate to increase with particle size [26].

As illustrated in Fig 8, the effects of different crushing processes on the abrasion resistance of RAP materials of diverse grain sizes was demonstrated. The findings of the study demonstrated that all four crushing methods can effectively enhanced the abrasion resistance of RAP materials. Specifically, the mean percentage reductions in abrasion values for hammer crushing, small mesh hammer crushing, jaw crushing, and double roller crushing were 23.87%, 21.97%, 13.16% and 5.74%, respectively. This result indicates that the process of hammer crushing significantly enhances the abrasion resistance of RAP materials. It is important to note that the reduction of the abrasion value of coarse aggregate is generally higher than that of fine aggregate. This indicates that the improvement of the abrasion resistance of coarse aggregate is more obvious when using refined processing technology. The underlying reason for this phenomenon is that the crushing process exerts a more substantial influence on the dissociation of coarse aggregate agglomerates, while the fine aggregate contains a greater quantity of residual asphalt, dust, and clay particles. Consequently, this results in a comparatively minor alteration in its abrasion performance post-crushing.

**Fig 8:**
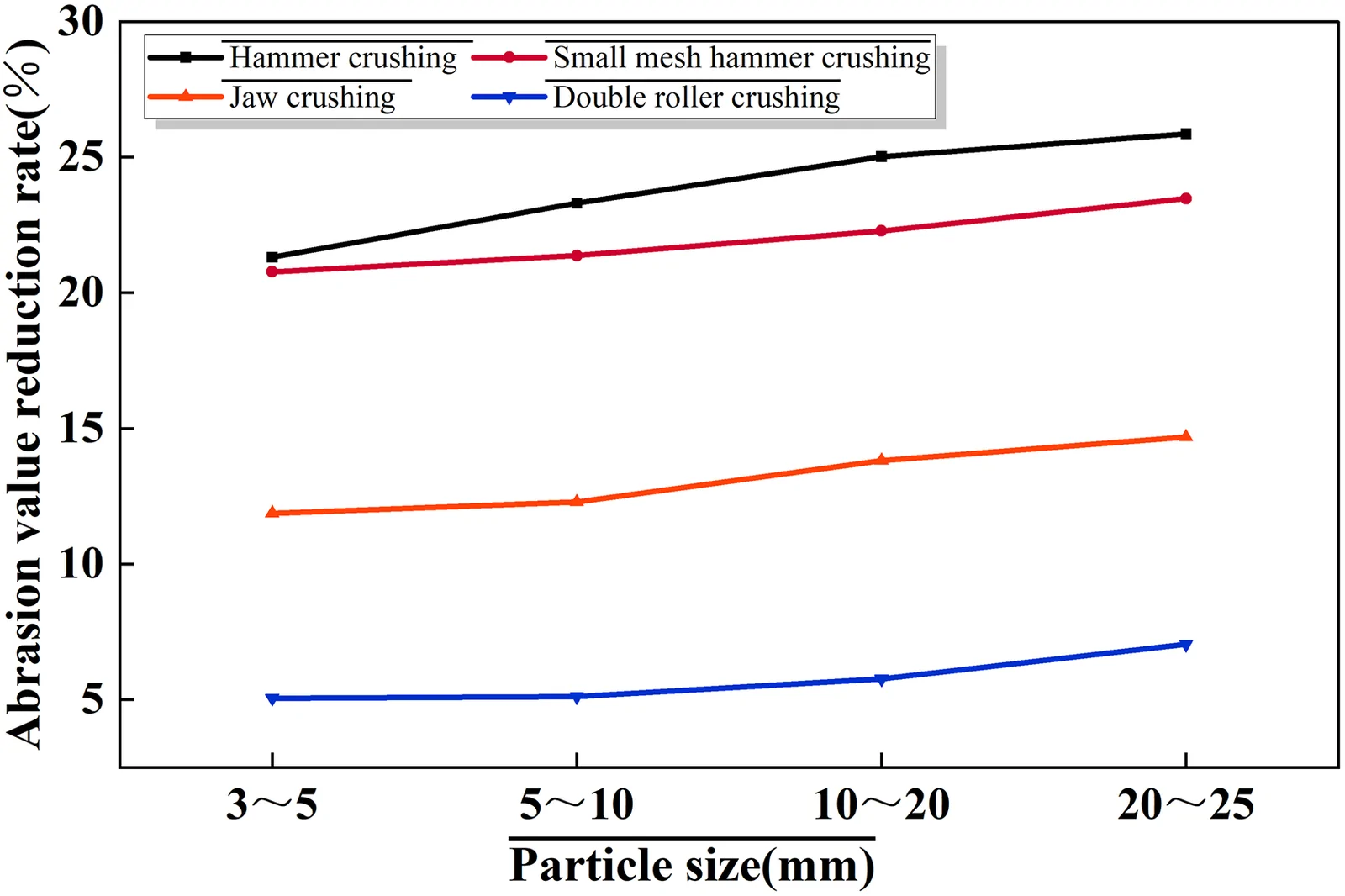
Reduction rate of abrasion value of RAP materials of various particle sizes after crushing.

#### 4.2.4 Content of needle and flake particles.

The needle and flake particle contents of RAP and natural aggregate are shown in Fig 9(a)–9(d).

**Fig 9:**
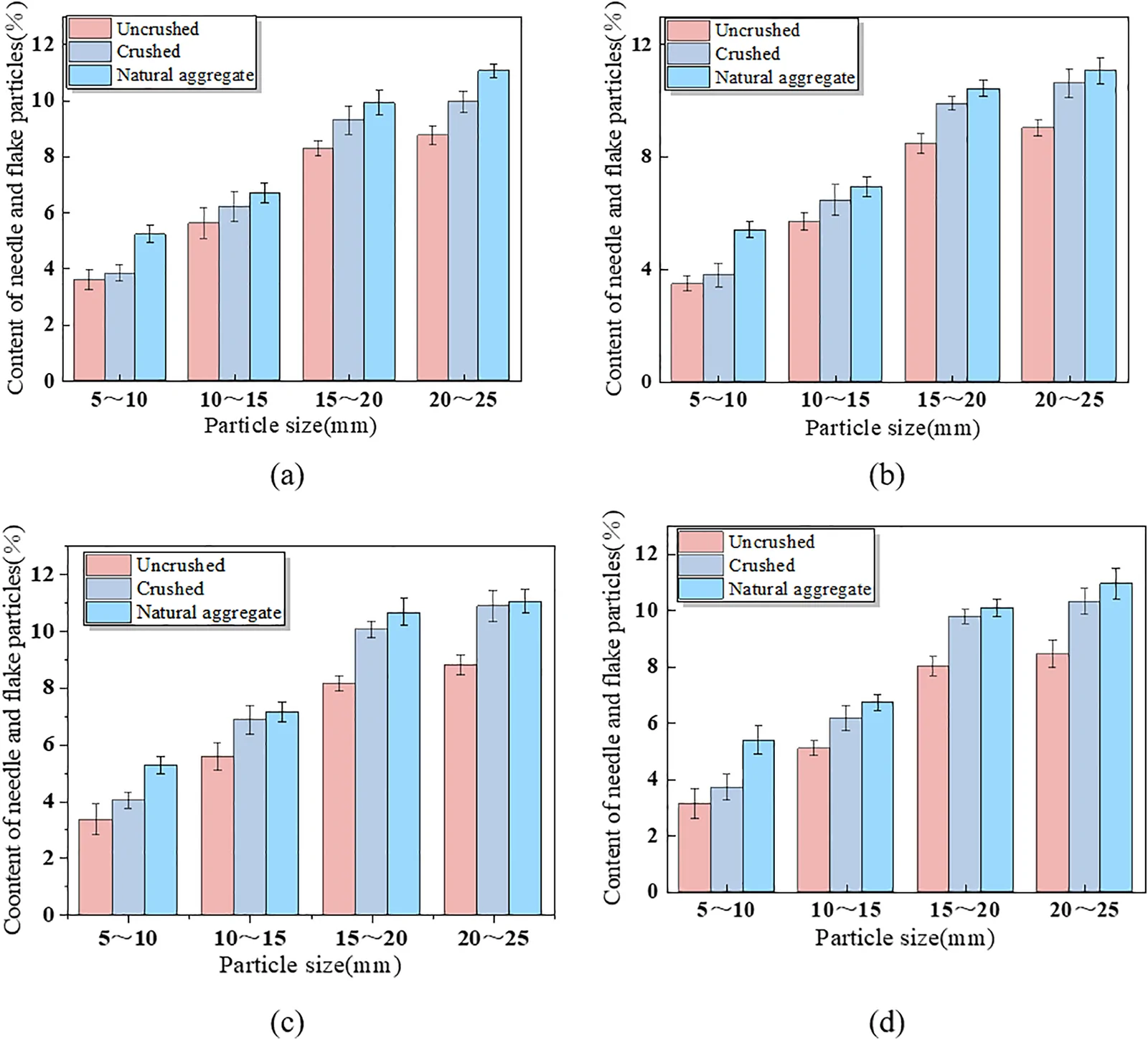
Changing law of content of needle and flake particles of aggregates of various grain sizes after crushing (a) Hammer crushing (b) Small mesh hammer crushing (c) Jaw crushing (d) Double roller crushing.

From Fig 9(a)–9(d), it can be seen that before and after the crushing process, the needle and flake particle contents of all RAP and natural aggregate fractions were below 20%, indicating that both untreated and pretreated RAP met the technical requirements for base layer construction, and the treated RAP could potentially substitute for natural aggregate. After the different crushing methods were applied, the needle-flake particle content of each RAP fraction increased, indicating that fine processing technology alters the morphological characteristics of the aggregate, making it thinner and finer. The increase in needle flake particle content after crushing is attributed to two mechanisms: (1) mechanical cleavage: Impact and shear forces create fresh fracture surfaces, producing more angular particles; and (2) agglomerate removal: Uncrushed RAP particles are often rounded due to the encapsulating asphalt mastic. The dissociation of these rounded agglomerates reveals the internal angular aggregates, thereby increasing the measured needle and flake particle content. While high needle and flake particle content can theoretically reduce breakage resistance, the values here (maximum <11%) remain significantly below the stringent limit of 15% required for high-grade highway base layers as specified in the *Technical Guidelines for Construction of Highway Roadbases* (JTG/T F20-2015). Therefore, this slight morphological change is considered an acceptable engineering trade-off to achieve the primary goal of binder stripping. The results indicated that the needle and flake particle content of both RAP materials and natural aggregates demonstrated an increasing trend with increasing particle size. The needle and flake particle content of RAP materials exhibited marked variations when subjected to diverse crushing processes. The range of the content post-hammer crushing was 3.83% ~ 9.95%, while small mesh hammer crushing yielded 3.8% ~ 10.63%, jaw crushing yielded 3.17% ~ 10.32%, and double roller crushing yielded 4.89% ~ 10.89%. It is noteworthy that following the application of the four crushing methods, the content of needle flake particles in each size of RAP aggregate was found to be significantly lower than the corresponding index of natural aggregate. This difference may be attributed to the fact that the crushing and screening of natural aggregates produce particles with more pronounced angularity and irregular shapes. In contrast, incomplete separation of asphalt and aggregate leaves more rounded agglomerates in RAP, resulting in a lower needle and flake particle content [27].

Fig 10 provides an analysis of the growth rate of needle and flake particle content of RAP material for each particle size after treatment by different crushing methods. The results presented in Fig 10 demonstrated a positive correlation between the growth rate of needle and flake particle content of RAP material after treatment by different crushing processes and the particle size. Specifically, the growth rate of needle and flake particle content demonstrated a continuous upward trend with the increase of RAP particle size. This phenomenon suggests that the crushing process had a more substantial influence on the morphological changes of large aggregates. The underlying reason for this phenomenon is that large aggregates are subjected to greater levels of mechanical friction and collision during the crushing process. This, in turn, results in the intensification of the degree of structural damage, thereby facilitating more effective decomposition of the original agglomerates. Conversely, the effect of small-sized aggregates on agglomerate decomposition is limited due to the relatively weak stress they exert. After the treatment of four crushing methods, hammer crushing increased the content of needle-like particles of each size by 6.69%, 10.5%, 12.45% and 13.71% respectively, which is lower than the growth rate of the other three crushing methods. This indicates that hammer crushing has the smallest effect on the content of needle-like particles of RAP materials of various particle sizes. After double roller crushing, the needle and flake particle content increased by 20.19%, 22.97%, 23.28%, and 23.25%, respectively, indicating that double roller crushing caused the greatest increase in the needle and flake particle content of RAP. In order to ensure that the strength of the recycled mixture meets the construction needs after mixing RAP material, aggregates with a lower content of needle flake particles should be used. Therefore, when selecting a processing method, the effects of different crushing methods on aggregate morphology should be considered, and RAP aggregates with lower needle and flake particle contents should be preferred [28].

**Fig 10:**
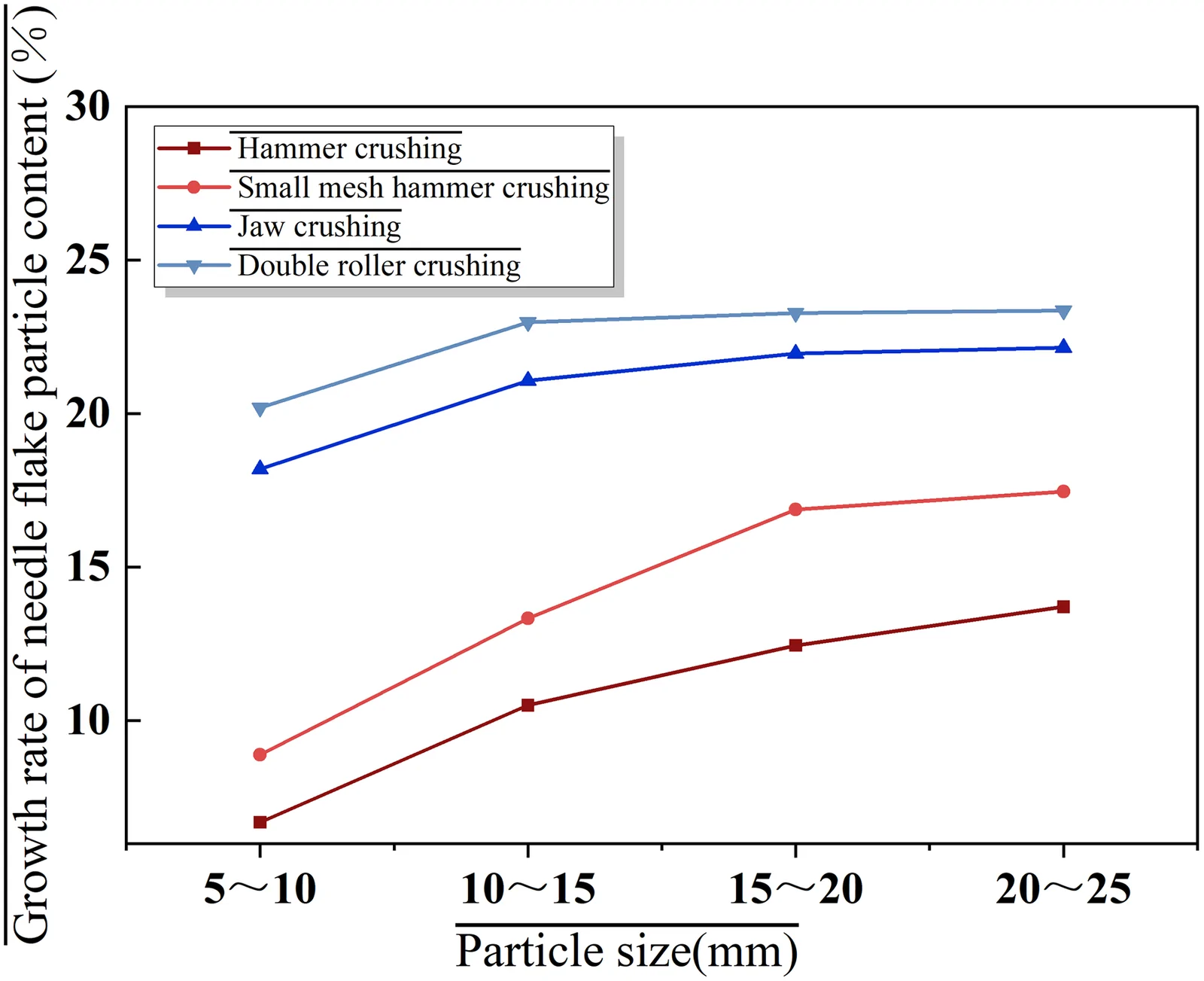
Growth rate of content of needle and flake particles of RAP materials of various particle sizes after crushing.

### 4.3 Effect of crushing on micro-morphology of RAP material

#### 4.3.1 SEM test results and analysis.

Fig 11 shows the SEM images of the limestone samples at magnifications of 5000−200. From Fig 11(a) and 11(b), it can be seen that the aggregate surface is rich in pores, there are a large number of individual particles in the size range of 2 ~ 10 μm, the particles are mainly flaky, the surface is dense and angular, and there are a large number of flaky secondary particles distributed between the pores. The particles in Fig 11 exhibit angular polyhedral structures with dense surfaces and no obvious signs of dissolution, indicating that they are dominated by mechanical crushing, and the integrity of mineral crystallization is high. From Fig 11(c) and 11(d), it can be seen that as the observation scale decreases, the particles in Fig 11(c) are interlocked through their uneven surface structures to form a multiscale adhesion unit. The rough surface and irregular particle distribution together form a three-dimensional skeleton structure, which enhances the internal friction resistance of the mixture. In addition, Fig 11(d) shows a microfracture network less than 2 μm wide extending along the particle boundaries. This network may provide stress transfer paths and have a dual effect on fracture toughness. It may also increase interparticle friction and promote the formation of an aggregate skeleton, thereby contributing to mixture strength.

**Fig 11:**
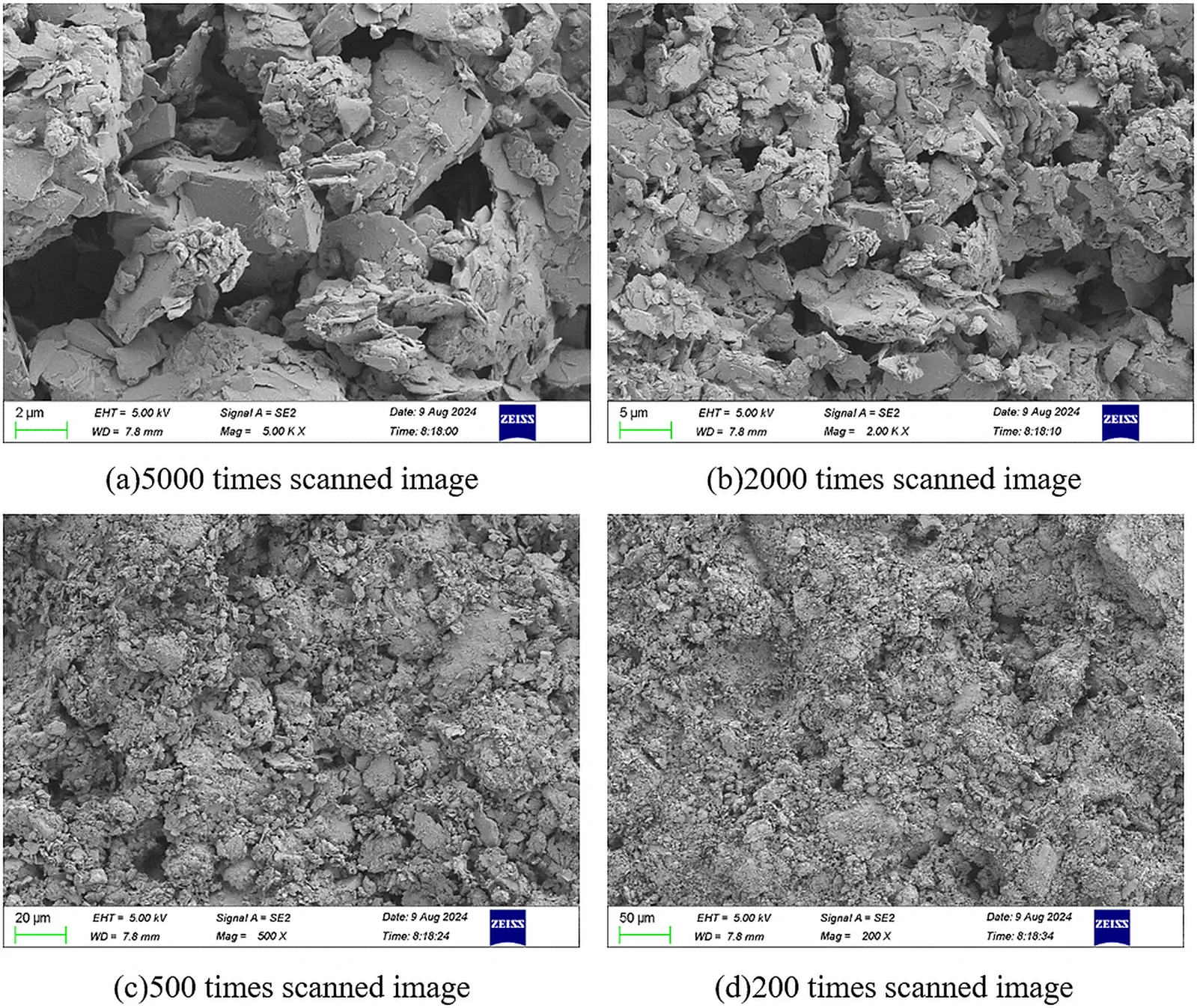
Microscopic morphology of limestone aggregates (a) 5000 times (b) 2000 times (c) 500 times (d) 200 times.

Fig 12 shows the micro-morphology of the uncrushed RAP sample. Compared to Fig 11, a bitumen film can be observed in addition to the rock material, and some rock particles adhered to the bitumen, making the surface smooth. It should be noted that the identification of bitumen was primarily based on its distinct micro-morphological characteristics—specifically its smooth, continuous film structure and tendency to form spherical clusters—which contrasts significantly with the angular, crystalline structure of the mineral aggregates and fine dust. The asphalt bound the rock particles into spherical clusters, resulting in fewer flaky particles and more spherical particles, so the morphology of the RAP material is predominantly spherical with a lower content of needle and flake particles than the natural aggregate. Comparison of Figs 11(c), 11(d), 12(c) and 12(d) shows that RAP had more and larger surface pores than natural aggregate. This is because the agglomeration phenomenon generates a new micro-morphology, resulting in a different pore size, number, and distribution state on the surface of the RAP material compared to the natural aggregate. Since RAP had more and larger pores than natural aggregate, the water absorption rate of natural aggregate is lower than that of RAP material. From the microstructure, it can be observed that adhesion between asphalt and aggregate formed numerous agglomerates, so the crushing value of RAP material is higher than that of natural aggregates, and the loading capacity of RAP material is weaker than that of natural aggregates [29].

**Fig 12:**
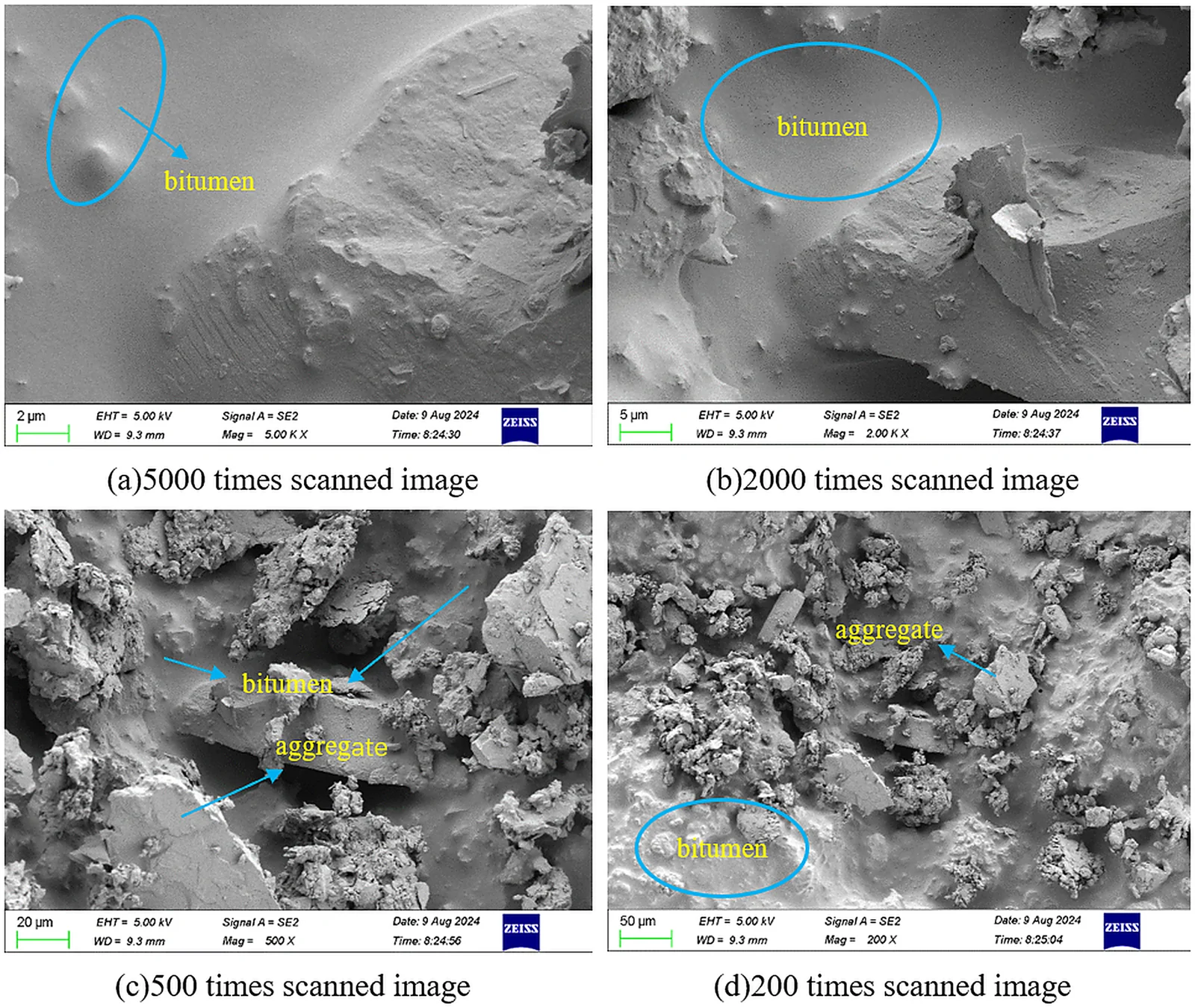
Microscopic morphology of uncrushed RAP (a) 5000 times (b) 2000 times (c) 500 times (d) 200 times.

As shown in Fig 13, the asphalt content on the surface of RAP was visibly reduced after fine separation and processing. Comparison of Fig 12(a) and 12(b) with Fig 13(a) and 13(b) shows that fewer asphalt films were present after crushing. The number of lamellar particles and particles in the 0–10 μm range increased, whereas the number of spherical particles decreased. Comparison of Fig 12(c) and 12(d) with Fig 13(c) and 13(d) shows that crushed RAP contained fewer particles in the 20–50 μm range than uncrushed RAP. Most particles in the 0–10 μm range were in contact and aggregated, whereas a small number of particles larger than 50 μm had dense and smooth surfaces. This occurred because crushing fractured some aggregate particles and altered their surface morphology. Compared with limestone in Fig 11, crushed RAP contained more particles larger than 50 μm, and these particles had smoother surfaces.

**Fig 13:**
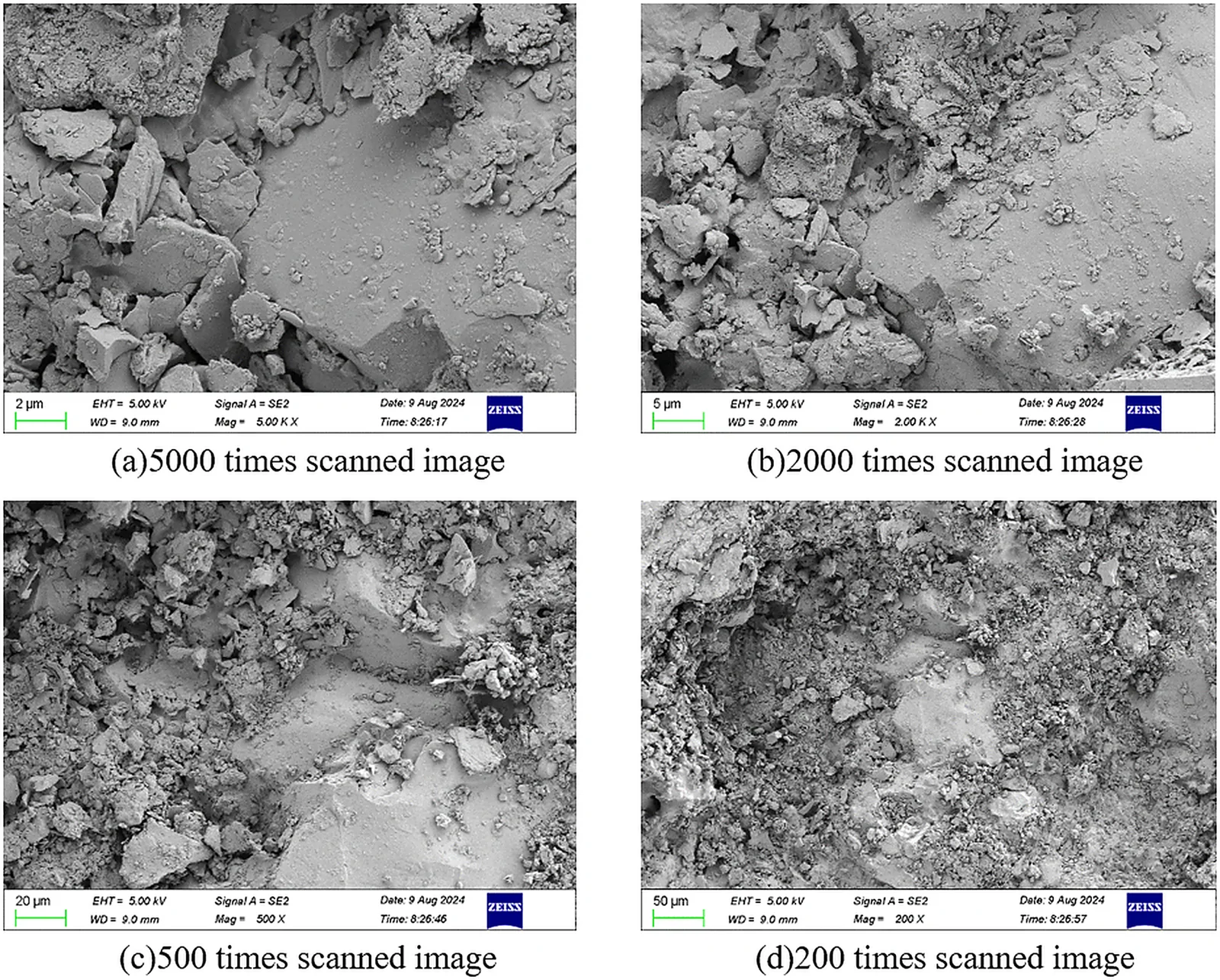
Microscopic morphology of crushed RAP (a) 5000 times (b) 2000 times (c) 500 times (d) 200 times.

#### 4.3.2 3D laser planar scanning test results and analysis.

The effects of different crushing methods on the roughness of each RAP particle size fraction are shown in Fig 14. From Fig 14(a)–14(d), the roughness values of limestone and RAP increased with particle size, and crushed RAP showed the same pattern. The surface texture of the uncrushed RAP material was found to be considerably smoother in comparison to that of the natural aggregate. This phenomenon is primarily attributed to the agglomeration effect, which drives the particle morphology to a spherical shape, thereby reducing the surface angular features and the number of needle-like particles. The roughness of the crushed 3–5 mm RAP fraction increased, whereas the roughness of both coarse RAP fractions decreased. This phenomenon suggests that the crushing process exerts a discernible polishing effect on the surface of the coarse aggregate. This decrease occurred because mechanical impact, shear, and compression removed adhered fine aggregates and aged asphalt from the surfaces of large RAP particles. Repeated friction also gradually wore the particle corners, resulting in lower surface roughness [30]. For particles smaller than 5 mm, stress concentration caused the harder mineral components to fracture suddenly, creating fresh irregular surfaces. These small-sized RAP materials are rapidly discharged without experiencing long-term friction, retaining sharp edges and rough structures.

**Fig 14:**
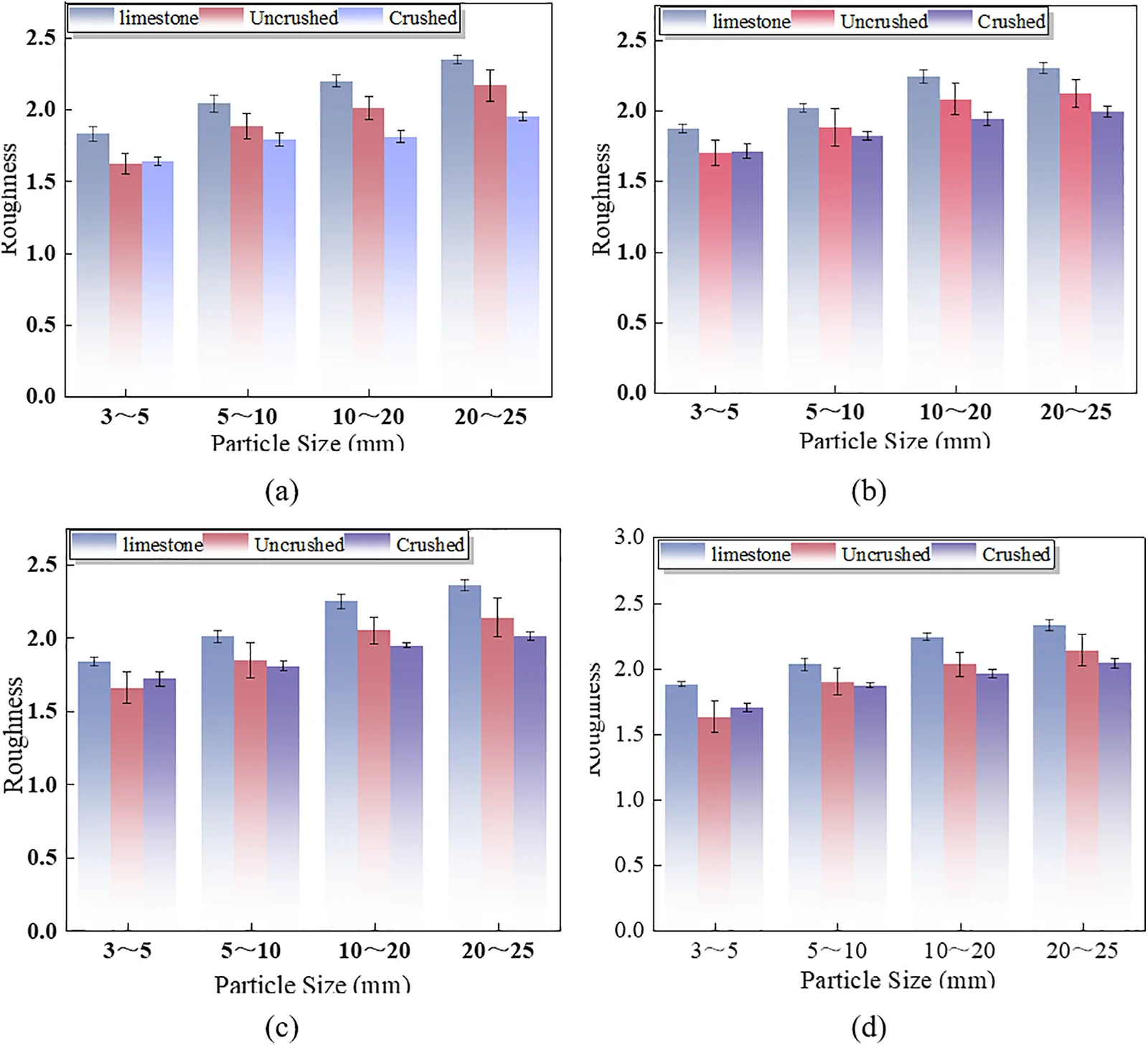
Aggregate roughness of each grain size before and after crushing (a) Small mesh hammer crushing (b) Hammer crushing (c) Jaw crushing (d) Double roller crushing.

From Fig 14(a)–14(d), it can also be seen that the roughness of 3 ~ 5 mm RAP materials increased by 0.02, 0.01, 0.06 and 0.07 after small mesh hammer crushing, hammer crushing, jaw crushing, and double roller crushing, respectively, because jaw crushing and double roller crushing promoted reagglomeration of fine aggregates, asphalt, and soil, producing uneven and rough surfaces. Small mesh hammer crushing and hammer crushing caused wear of the aggregates smaller than 5 mm, resulting in an increase in the roughness of the RAP materials of 3 ~ 5 mm size after different crushing methods. There is a significant difference in the reduction of surface roughness of RAP materials with grain sizes above 5 mm after treatment by different crushing processes. Specifically, small mesh hammer crushing reduced the roughness by 0.09 ~ 0.22, hammer crushing by 0.06 ~ 0.13, jaw crushing by 0.04 ~ 0.12, and double roller crushing by 0.03 ~ 0.10, which suggests that the small mesh hammer crushing process shows the optimal effect in reducing the roughness of the surface of the RAP coarse aggregates.

### 4.4 Comparison with prior studies

To contextualize the effectiveness of the evaluated crushing methods, it is necessary to compare these results with existing RAP processing technologies. Qiu et al. [12] utilized foaming technology to achieve up to 90% warm re-use of porous asphalt. While their approach focused on binder rejuvenation, this study demonstrates that comparable high-efficiency separation, reaching up to a 96.6% residual mass ratio for coarse particles, can be achieved through purely mechanical impact methods utilizing the small mesh hammer without thermal or chemical additives. Furthermore, Li et al. [14] reported that centrifugal crushing reduced the agglomeration of 5–15 mm particles to below 21.6%. In comparison, the impact based hammer method used in this study yielded residual mass ratios between 77.8% and 90.4% for similar size ranges. This variance is fundamentally due to the differing mechanisms: centrifugal processing utilizes rotational inertia and friction, whereas hammer crushing applies direct, high-energy kinetic impact, which is more aggressive in stripping adhered mastic.

Regarding aggregate integrity, Xu et al. [16] found that while flexible-rigid coupled separation reduced agglomeration, it also led to a decrease in the dynamic stability of the mixtures. Conversely, the mechanical property tests in this study confirm that hammer crushing significantly reduces both crushing and abrasion values, effectively bringing the mechanical integrity of the RAP aggregates closer to that of natural limestone. Finally, Ai et al. [15] demonstrated that asphalt mixtures containing 40% RAP treated via rotary decomposition meet performance specifications. These findings extend the previous results by showing that impact-based crushing can achieve even higher deagglomeration efficiencies, potentially allowing for greater RAP incorporation rates in highway base layers.

### 4.5 Relationship between RAP material agglomeration and aggregate properties

Based on test data including residual mass ratio, water absorption, crushing value, abrasion value, needle and flake particle content, and surface roughness, quantitative relationship models were established using linear regression. The detailed regression equations and coefficients of determination (R²) for each property are summarized in Table 4. Fig 15 presents the fitted curves along with the corresponding model equations and key parameters such as R².

**Table 4:** Regression parameters relating RAP residual mass ratio to aggregate properties.

Dependent Variable	Linear Regression Model	R²
Water Absorption	y = 1.7307x - 2.1537	0.9013
Crushing Value	y = −0.5158x + 10.6668	0.9215
Abrasion Value	y = −0.1026x + 2.9789	0.7771
Flat elongated particles Content	y = 0.4591x - 3.1005	0.7659
Roughness	y = −2.9241x + 6.3742	0.7414

The data obtained from the four crushing methods followed the same overall trends, suggesting that the observed correlations were not dominated by a single equipment type.

**Fig 15:**
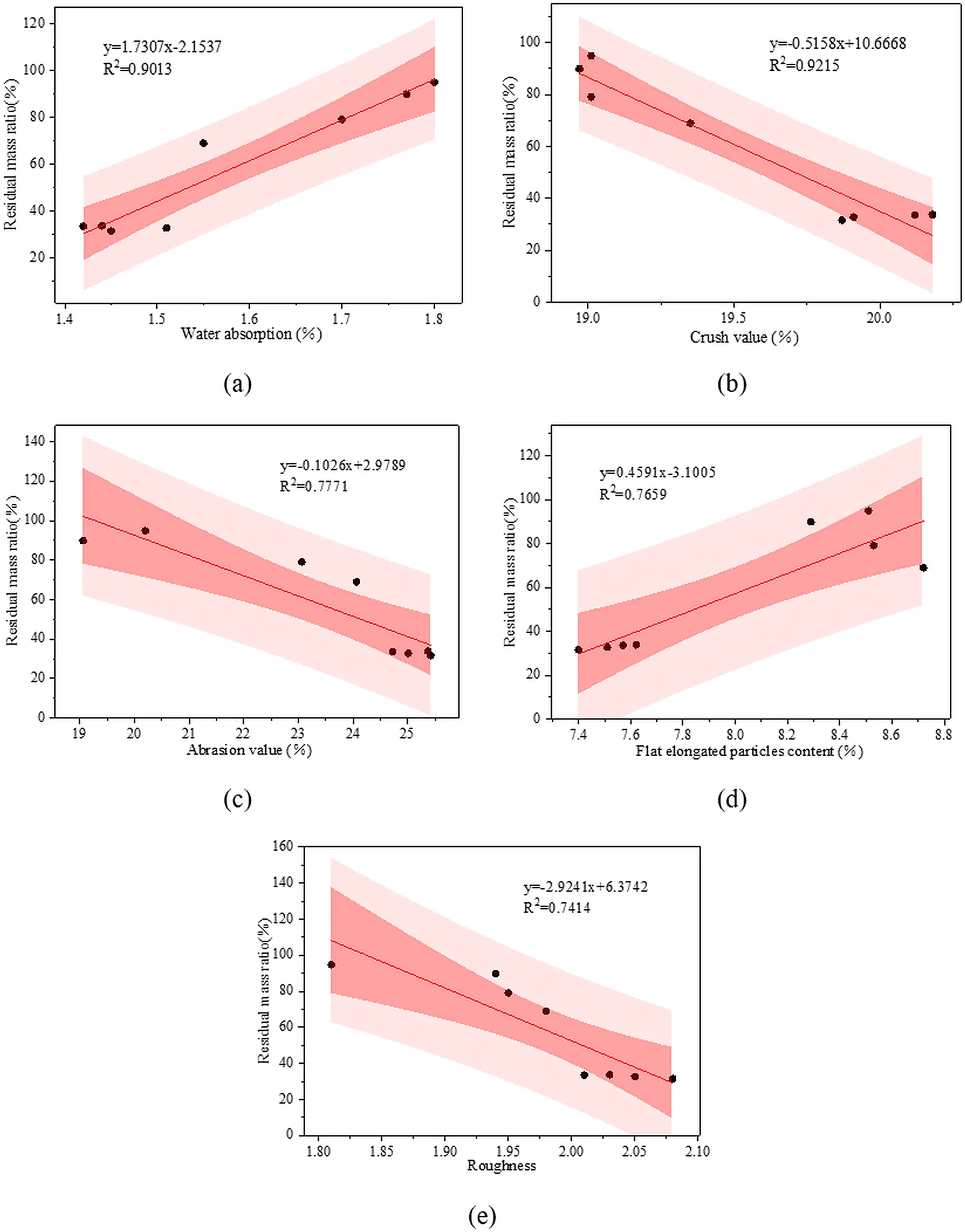
Relationship between RAP material agglomeration and aggregate properties (a) Water absorption rate (b) Crushing value (c) Abrasion value (d) Content of needle and flake particles (e) Roughness.

The results in Fig 15(a)–15(e) show that the residual mass ratio of RAP was significantly correlated with water absorption, crushing value, abrasion value, needle and flake particle content, and surface roughness. The crushing process affected RAP properties in two ways. The crushing value, abrasion value, and surface roughness were negatively correlated with the residual mass ratio, whereas water absorption and needle and flake particle content were positively correlated with the residual mass ratio [31].

The RAP material crushing value had the highest correlation with the residual mass ratio, with R^2^ value of 0.9215, and the abrasion value also showed a high correlation. This strong linear correlation is not coincidental but reveals an inherent physical coupling between the two core outcomes of the crushing process: asphalt stripping efficiency and aggregate damage. Both outcomes are directly driven by the kinetic energy input from the crushing equipment. As the crushing intensity increases, a determinate proportional relationship emerges between the energy consumed to overcome bitumen–aggregate interfacial adhesion, which increases the residual mass ratio, and the energy transmitted into the aggregate interior that propagates micro-cracks and increases the crushing value. Therefore, this linear relationship quantitatively characterizes the inevitable engineering trade-off between cleaning efficiency and aggregate structural integrity. The slope of this line can be regarded as a characteristic signature of the energy distribution for a specific crushing mode. Regarding the surface characteristics, due to the decomposition of RAP aggregates after crushing, the aged asphalt was peeled off from the aggregate surface, and the rough surface originally bonded by asphalt was changed to a relatively smooth surface closer to the original aggregate [32]. This morphological transformation provides a clear mechanistic explanation for the negative correlation observed between surface roughness and the residual mass ratio. Unlike natural aggregates where roughness typically enhances interlocking, the high roughness in RAP is primarily a signature of adhered aged asphalt mastic. Consequently, the crushing-induced reduction in roughness signifies the effective stripping of this cohesive mastic layer, indicating a cleaner and more deagglomerated particle state. However, it is important to emphasize that these regression models establish statistical correlations rather than definitive causality. While the high R^2^ values indicate strong predictive relationships, future studies with larger sample sizes are recommended to further validate the robustness of these equations.

Finally, the coefficients of determination for the relationships of needle and flake particle content and water absorption with the residual mass ratio were 0.7659 and 0.9013, respectively. This positive correlation is attributed to the mechanical nature of crushing: as agglomerates are effectively broken down to increase the residual mass ratio, closed pores are reopened, which increases water absorption, and the mechanical splitting action inevitably produces more angular and flaky particles [33]. Therefore, the crushing value and the residual mass ratio can be used as the key control indices for the crushing process of RAP material.

While the high coefficient of determination (R^2^ > 0.7) indicates a strong correlation between the residual mass ratio and aggregate properties, it is acknowledged that the assessment of bedrock or aggregate spatial distribution often involves uncertainties. Recent studies have highlighted the importance of calculating Confidence Intervals (CI) and Prediction Intervals (PI) to evaluate the reliability of predictive models, especially when dealing with indirect measurements or complex spatial variability [34]. Although the current study relies on linear regression under controlled laboratory conditions, future research involving larger datasets and field variability could benefit from incorporating PI and CI analyses to further quantify the prediction bounds and model robustness.

### 4.6 Limitations and model applicability

The quantitative relationship models established in this study (Fig 15) are based on experimental data from a single source of RAP material and a limited number of samples (n = 8 for each test group). While the high coefficients of determination (R² > 0.7) indicate strong correlations under the tested conditions, the generalizability of these models to RAP materials from different sources, regions, or aging conditions may be limited. Similar to findings in geotechnical predictive modeling [35], regression models based on larger and more diverse datasets tend to exhibit greater robustness and wider applicability. Therefore, the proposed correlations should be interpreted as indicative trends rather than universal predictive equations. Future studies incorporating multi-source RAP materials and expanded sample sizes are recommended to validate and refine these relationships.

### 4.7 Scale-up considerations

The physical property improvements observed in this study were achieved under controlled laboratory conditions. Scaling these processes to industrial plant conditions introduces several variables that may alter the crushing effects. First, industrial crushers operate at significantly higher continuous throughput rates, which reduce the specific energy input per particle and may lower the overall deagglomeration efficiency compared to laboratory batch processing. Second, the continuous feeding mechanisms in industrial operations create complex particle-bed interactions. In such environments, compressive forces are transferred through multiple particles simultaneously, which differs fundamentally from the discrete, single-particle impacts simulated in laboratory devices. Additionally, variability in field moisture content and stockpile temperatures can significantly influence the rheological behavior of the aged binder during crushing. Consequently, pilot-scale and full-scale field validation trials are highly recommended before directly applying these laboratory findings to industrial plant designs.

### 4.8 Practical engineering implications

The selection of crushing technology in practical engineering must balance performance benefits with operational costs. While the laboratory results indicate that small mesh hammer crushing achieves the highest deagglomeration efficiency and superior aggregate properties, this impact-based method (operating at 1300 rpm) typically involves higher energy consumption and equipment wear compared to compression based methods such as jaw or roller crushing (operating at <300 rpm). Consequently, for high-specification applications where aggregate angularity and cleanliness are paramount, hammer crushing is recommended. Conversely, for general base layer construction where cost efficiency is prioritized, jaw or roller crushing offers a viable, lower energy alternative. This trade-off suggests that the choice of equipment should be tailored to the specific quality requirements and economic constraints of the road project.

It is also important to consider the influence of aggregate lithology. The RAP material used in this study was derived from limestone, which typically has lower hardness and crushing resistance than igneous rocks such as granite or basalt. Consequently, the high degree of aggregate degradation manifested as an increase in needle and flake particle content under hammer crushing likely represents a worst-case scenario. In practical applications involving harder lithologies, the impact-induced degradation is expected to be less severe. Therefore, while the relative performance ranking of the crushing methods remains valid, the absolute degradation values reported in this study should be interpreted as a conservative baseline for softer aggregates.

## 5. Conclusion

In this study, a range of crushing techniques were employed for the pretreatment of RAP material, including small mesh hammer crushing, hammer crushing, jaw crushing, and double roller crushing. The impact of these processes on the degree of agglomeration, physical and mechanical properties, and microstructure of the RAP material was analyzed. Additionally, the relationship between the agglomeration of the RAP material and aggregate properties was determined. The main conclusions are as follows:

(1) The quantitative correlation model showed that the coefficient of determination (R²) exceeded 0.7 for all performance indicators. The model indicates that reductions in RAP crushing value, abrasion value, and surface roughness reflect successful deagglomeration, whereas asphalt stripping inevitably increases the needle and flake particle content and water absorption.
(2) The small mesh hammer crushing process exhibited the highest efficiency in dissociating RAP agglomerates, yielding residual mass ratios of 77.8%–96.6% across all particle sizes, significantly outperforming the hammer, jaw, and double roller methods. Furthermore, the effectiveness of the deagglomeration treatment increased as the initial RAP particle size increased.
(3) While all crushing treatments enhanced the mechanical properties of RAP, hammer-based methods were the most effective in reducing the crushing and abrasion values to levels closer to those of natural aggregates. It is acknowledged that the intensive impact action caused greater aggregate degradation and dust generation, which represents a form of material waste; however, from a practical and economic perspective, this is justified by the significant improvement in RAP quality. Consequently, despite the notable increase in water absorption and a slight rise in needle and flake particle content due to the exposure of fresh surfaces, all indicators remained well within the technical specifications for highway base layers.
(4) Micro-morphological evaluations qualitatively validate the physical findings. SEM and 3D laser scanning confirm that impact crushing effectively removes adhered asphalt films and significantly reduces the surface roughness of coarse aggregates by 0.09 to 0.22 under small mesh hammer crushing. Due to the limited number of particles analyzed, these observations provide mechanistic evidence rather than statistical characterization.

While this study relied on macroscopic water absorption and SEM observations of surface morphology to characterize pore structure evolution, future research could utilize X-ray Computed Tomography (XCT) to quantitatively analyze the internal pore connectivity and 3D void distribution of the crushed aggregates. Future work should also include mechanical testing of the compacted mixtures, such as shear box or triaxial tests, to validate how the improved aggregate angularity translates to the macroscopic shear strength of the pavement base layer. Future research should employ a multi-source, large-sample approach to validate and generalize the relationships established in this study, thereby enhancing the predictive reliability for recycled pavement materials. Finally, pilot-scale and full-scale field validation trials are recommended to confirm these laboratory mechanisms under continuous industrial operating conditions.
